# Plasma proteins related to inflammatory diet predict future cognitive impairment

**DOI:** 10.1038/s41380-023-01975-7

**Published:** 2023-02-03

**Authors:** Michael R. Duggan, Lauren Butler, Zhongsheng Peng, Gulzar N. Daya, Abhay Moghekar, Yang An, Stephen R. Rapp, Kathleen M. Hayden, Aladdin H. Shadyab, Ginny Natale, Longjian Liu, Linda Snetselaar, Ruin Moaddel, Casey M. Rebholz, Kevin Sullivan, Christie M. Ballantyne, Susan M. Resnick, Luigi Ferrucci, Keenan A. Walker

**Affiliations:** 1 Laboratory of Behavioral Neuroscience, National Institute on Aging, Baltimore, MD, USA; 2 Department of Neurology, Johns Hopkins University School of Medicine, Baltimore, MD, USA; 3 Department of Social Sciences and Health Policy, Wake Forest University School of Medicine, Winston-Salem, NC, USA; 4 Department of Psychiatry & Behavioral Medicine, Wake Forest University School of Medicine, Winston-Salem, NC, USA; 5 Herbert Wertheim School of Public Health and Human Longevity Science, University of California, San Diego, La Jolla, CA, USA; 6 Program in Public Health, Stony Brook University School of Medicine, Stony Brook, NY, USA; 7 Department of Epidemiology and Biostatistics, Drexel University Dornsife School of Public Health, Philadelphia, PA, USA; 8 Department of Epidemiology, University of Iowa, Iowa City, IA, USA; 9 Laboratory of Clinical Investigation, National Institute on Aging, Baltimore, MD, USA; 10 Department of Epidemiology, Johns Hopkins Bloomberg School of Public Health, Baltimore, MD, USA; 11 Department of Medicine, University of Mississippi Medical Center, Jackson, MS, USA; 12 Section of Cardiovascular Research, Department of Medicine, Baylor College of Medicine, Houston, TX, USA; 13 Translational Gerontology Branch, National Institute on Aging, Baltimore, MD, USA

## Abstract

Dysregulation of the immune system and dietary patterns that increase inflammation can increase the risk for cognitive decline, but the mechanisms by which inflammatory nutritional habits may affect the development of cognitive impairment in aging are not well understood. To determine whether plasma proteins linked to inflammatory diet predict future cognitive impairment, we applied high-throughput proteomic assays to plasma samples from a subset (*n* = 1528) of Women’s Health Initiative Memory Study (WHIMS) participants (mean [SD] baseline age, 71.3 [SD 3.8] years). Results provide insights into how inflammatory nutritional patterns are associated with an immune-related proteome and identify a group of proteins (CXCL10, CCL3, HGF, OPG, CDCP1, NFATC3, ITGA11) related to future cognitive impairment over a 14-year follow-up period. Several of these inflammatory diet proteins were also associated with dementia risk across two external cohorts (ARIC, ESTHER), correlated with plasma biomarkers of Alzheimer’s disease (AD) pathology (Aβ_42/40_) and/or neurodegeneration (NfL), and related to an MRI-defined index of neurodegenerative brain atrophy in a separate cohort (BLSA). In addition to evaluating their biological relevance, assessing their potential role in AD, and characterizing their immune-tissue/cell-specific expression, we leveraged published RNA-seq results to examine how the in vitro regulation of genes encoding these candidate proteins might be altered in response to an immune challenge. Our findings indicate how dietary patterns with higher inflammatory potential relate to plasma levels of immunologically relevant proteins and highlight the molecular mediators which predict subsequent risk for age-related cognitive impairment.

## INTRODUCTION

Elevated levels of inflammation, as well as immune dysregulation itself, can have deleterious effects on brain structure and function in aging [1, 2]. Accordingly, pro-inflammatory dietary patterns may influence the risk for cognitive decline by promoting both transient and chronic systemic inflammation marked by higher inflammatory signaling across multiple tissues [3, 4]. To quantify the inflammatory potential of an individual’s diet, researchers have increasingly utilized a Dietary Inflammatory Index (DII) that captures intake of various parameters (foods, nutrients, spices, etc.) and is positively correlated with select markers of inflammation, including interleukin-6 (IL-6) and tumor necrosis factor (TNF) [5–10]. Across multiple large cohorts, including the Women’s Health Initiative Memory Study (WHIMS), a pro-inflammatory diet (i.e., high DII score) has been associated with lower cognitive performance and higher risk of cognitive impairment and dementia [11–15]. However, it remains unknown how an inflammatory diet relates to an array of inflammatory and immune signaling proteins, as well as which of these molecular mediators may be associated with risk for future cognitive impairment, including dementia. Understanding how inflammatory diet is related to the immune proteome will provide novel insights into the mechanisms through which diet—a lifestyle factor and potential target for intervention—may influence brain health in older adults.

Using plasma samples from a subset of WHIMS participants along with state-of-the-art proteomic profiling, the present study first examined the association between inflammatory nutritional patterns and a comprehensive panel of inflammatory/immune proteins in women who were cognitively unimpaired at baseline. After identifying 55 proteins linked to inflammatory diet, we identified a subset of these inflammatory/immune proteins that were also associated with risk for incident cognitive impairment over a 14-year follow-up period. We demonstrate that several inflammatory diet proteins associated with cognitive impairment in WHIMS were also associated with dementia risk in two external cohorts, correlated with biomarkers of Alzheimer’s disease (AD) pathology (Aβ_42/40_) and/or neurodegeneration(NfL), and related to neurodegeneration-specific patterns of brain atrophy in a separate cohort. Additionally, we used publicly available RNA-seq results to demonstrate that the in vitro regulation of genes encoding these proteins may be altered in response to an immune challenge.

## METHODS

### Study sample

WHIMS participants were recruited from 39 sites of the larger Women’s Health Initiative (WHI) study (enrollment: 1996–99) [16–22]. Cognitive status was assessed on an annual basis [14] until the final follow-up assessment in 2018 for the current sample. At baseline, blood samples were collected and stored for later analysis, and participants completed the WHI Food Frequency Questionnaire (WHI-FFQ; based on the Block FFQ) [21, 23–26]. Participants were eligible for inclusion if they had more than one follow-up cognitive examination, completed a baseline WHI-FFQ and had available proteomic data from a WHIMS ancillary study. Participants were excluded if they were cognitively impaired at baseline or if all proteomic measurements failed QC (detailed below). Additional description of the study sample and the WHIMS ancillary study is provided in the Supplementary Methods.

### Energy-Adjusted Dietary Inflammatory Index (EDII)

Using responses from the WHI-FFQ to measure food components weighted by their inflammatory potential according to the Dietary Inflammatory Index (DII) [5–10, 14], we characterized each participant’s diet on a continuum from anti-inflammatory to pro-inflammatory, with a higher score indicating a more pro-inflammatory diet. Standardized dietary intake estimates were converted to centered percentiles for each component, multiplied by the corresponding component-specific inflammatory effect scores and summed to obtain overall scores, which were then adjusted for energy consumed per 1000 kcal (EDII); total energy intake was derived from the WHI-FFQ [27–30]. A full explanation of the EDII, its construct validation and its calculation are provided in the Supplementary Methods.

### Olink protein measurement

Proteins were measured with the Olink Inflammation and Immune Response 96-panels (a full list of proteins in each panel is provided in Supplementary Table 1) using high-throughput, proximity extension assays (PEA) from blood samples collected at baseline and stored at −80 °C using standardized protocols until the day of analysis [31, 32]. In brief, these assays use epitope-specific binding and hybridization of paired oligonucleotide antibody probes, which are subsequently amplified using qPCR, resulting in log base-2 normalized protein expression values. The median intraclass correlation (ICC) calculated using 82 blind duplicates was 0.93 and 0.91 for proteins on the Inflammation and Immune Response panels, respectively. Four proteins were measured in duplicate due to replicate probes on each panel (*IL5, IL6, IL10, CCL11*); after confirming duplicated proteins were highly correlated (*r*’s ≥ 0.88), one measure of each protein was randomly selected for subsequent analyses. We excluded samples that did not meet Olink quality control standards (*n* = 78). Additionally, 29 proteins were excluded from analyses due to >25% of samples either below protein-specific lower limits of detection (LOD) or failing QC (Supplementary Table 2). For the remaining proteins, values below LOD were imputed with LOD/2; the number of imputed values per protein varied (Supplementary Table 3) [33, 34]. Protein values beyond 5 SDs from the mean were winzorized. A total of 151 proteins were included in the analyses.

### Immune/Inflammatory pathways

Proteins were classified a priori into 11 functional categories relevant to immune biology, as described previously [35]. Protein classification was dependent on Gene Ontology (GO) annotations for a given pathway, and composite scores were calculated by taking the mean of the standardized values of proteins annotated in each pathway. A full list of proteins annotated for each pathway is provided in Supplementary Table 4.

### Cognitive assessment

Using standard neuropsychological tests coupled with central adjudication by a panel of expert clinicians, participants were classified as having MCI [36] or probable dementia [37] (Cognitively Impaired; CI) and no impairment (Cognitively Unimpaired; CU), as described previously [14]. A detailed description of the procedures used for cognitive assessment is provided in the Supplementary Methods.

### External validation of protein-dementia associations

Two external cohorts were used to validate protein associations with cognitive impairment. We used previously published results from the Epidemiological Study on the Chances of Cure, Early Detection and Optimized Therapy of Chronic Diseases in the Elderly Population (ESTHER) [34], which utilized blood samples drawn from the years 2000–02 to measure proteins using the same Olink Inflammation 96-panel used in WHIMS. After this blood draw, ESTHER participants were followed for approximately 17 years, during which time incident dementia was captured. Olink proteins associated with incident dementia were used to derive protein-specific odds ratios for dementia risk. We also used data from the Atherosclerosis Risk in Communities (ARIC) Neurocognitive Study as part of a cross-platform validation of protein-dementia associations. In ARIC, the SomaScan (version 4.0) proteomic platform [38] was applied to blood samples drawn from the years 2011–13 (study visit 5). Follow-up visits to identify incident dementia occurred through the end of 2019 (study visit 7). We related candidate proteins (defined below), measured using SomaScan, to time to dementia onset over an eight-year follow-up period. A full description of the ARIC study is provided in the Supplementary Methods.

### Amyloid and neurodegeneration biomarker measurement

Biomarkers of AD pathology (Aβ_42/40_) and neurodegeneration (NfL) were measured in EDTA plasma collected at baseline using the Single Molecule Array (Simoa) Neurology 4-Plex E (N4PE) assay on the Simoa HD-X instrument (Quanterix Corporation). Assays were run in duplicate and values averaged; ICCs were 0.89, 0.92, and 0.95 for Aβ_40_, Aβ_42_, and NfL, respectively. Aβ_42/40_ ratio was used in analyses; biomarker values were standardized and values beyond 5 SDs from the mean were winzorized.

### MRI-defined brain atrophy

To relate candidate proteins to MRI-defined neurodegeneration, we used data from the Baltimore Longitudinal Study of Aging (BLSA). Participants with brain volume measurements collected with 3 T MRI scans and available SomaScan (version 4.1) proteomic data were included [39–42]. We related candidate proteins to a machine-learning based index known as the Spatial Pattern of Atrophy for Recognition of Alzheimer’s Disease (SPARE-AD), which captures multi-variate changes in brain structure that have been validated to accurately discriminate cognitively normal subjects from other neurodegenerative phenotypes, particularly AD [43–49]. Detailed explanations of the BLSA and MRI procedures are provided in the Supplementary Methods.

### Gene expression analyses

For comparative gene expression analyses across immune-related tissues, we used consensus transcript expression levels (normalized Transcripts per Million; nTPM) from the Human Protein Atlas (HPA) and Genotype-Tissue Expression (GTEx) project. For comparative gene expression analyses across immune-related cell types, we used nTPM values in the HPA scRNA-seq dataset. Full descriptions of expression datasets are available online (https://www.proteinatlas.org/about/).

### Immune challenge (LPS) expression data

To assess the expression of genes coding for candidate proteins in immune-related cell types following an immune challenge, we searched literature databases for publications that utilized RNA-seq analyses in conjunction with in vitro immune cell types treated with lipopolysaccharide (LPS), an outer membrane component of Gram-negative bacteria that triggers immune cell responses [50]. Differential expression (decrease/ increase/no change) in response to LPS treatment was defined according to adjusted *p* values (<.05) reported in each respective study; in cases where adjusted *p* values were not reported, differential expression was defined by ≥ 3.0 log_2_ fold change.

### Protein characterization

To understand the biological implications and functional relevance of candidate proteins, we used a variety of complementary, open-source databases and analytic tools. A detailed description of the methods used for protein characterization is provided in the Supplementary Methods.

### Covariates

Covariates were measured or self-reported at enrollment and included age, WHI hormone therapy arm (drug/placebo), recruitment region, education level, *APOE*ε4 carrier status (0/ ≥ 1 ε4 alleles), hypertension, diabetes, obesity (BMI ≥ 30), and estimated glomerular filtration rate (eGFR)-creatinine. eGFR was calculated based on serum creatinine and demographic characteristics (age, sex, race) using the CKD-EPI equation [51]. Race and sex were not included as covariates because all participants in the current analyses were white and female.

### Statistical analysis

We used partial Spearman correlations (adjusted for age) to relate proteomic variables (individual proteins, pathway composite scores) to EDII scores, DII scores, and age. Subsequently, multivariable logistic regression models were used to examine associations of EDII-related proteomic variables with odds of cognitive impairment, adjusting for each of the demographic, physiological, and cardiovascular risk factors listed in the Covariate section (primary model). Unadjusted models were also examined, as were sensitivity analyses that examined the effect of additionally adjusting for lifestyle factors (smoking, alcohol use, sleep quality, physical function). Next we extracted results from the ESTHER cohort, which used the same Olink Inflammation panel to examine the relationship between inflammatory proteins and 17-year dementia risk [34]. We pooled the ESTHER and WHIMS cohort-specific effect estimates using a fixed-effect, inverse variance-weighted meta-analysis to enhance the accuracy and generalizability of our results [52, 53]. We then leveraged data from another external cohort (ARIC) to validate the association between individual proteins and eight-year dementia risk using Cox proportional hazards regression models adjusted for age, sex, race-center, education, *APOE*ε4, eGFR-creatinine, and cardiovascular risk factors (BMI, diabetes, hypertension, and current smoking status). Statistical significance was defined at two-sided *p* < 0.05. All statistical analyses were performed in R, version 3.4.1 (R Foundation). A more detailed description of our statistical analyses, including analyses used in secondary validation procedures, is provided in the Supplementary Methods

## RESULTS

### Sample characteristics

**Fig. 1:**
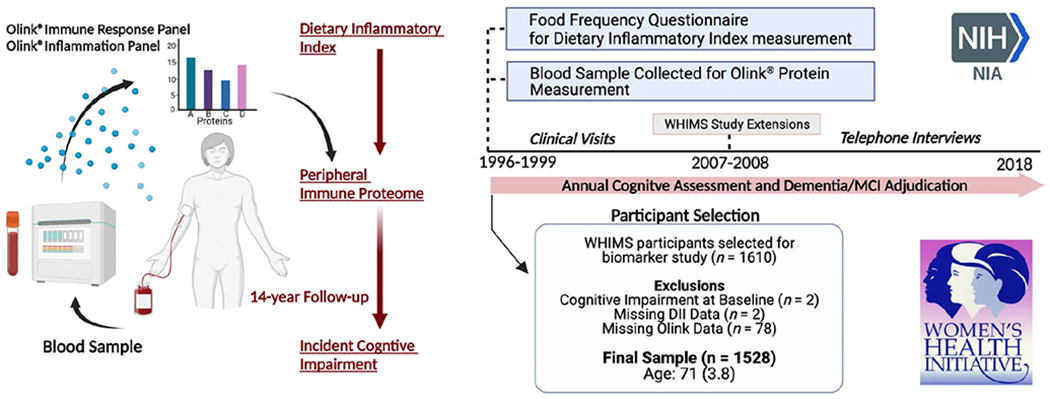
Study design and participant selection. Olink^®^ Inflammation and Immune Response Panels were applied to blood samples collected at baseline for WHIMS participants. The study sample was recruited from the larger Women’s Health Initiative (WHI) study, where enrollment began in 1996– 1999. Participants continued annual cognitive assessments in-person until 2007–08, when the study was extended and cognitive assessments were continued via telephone. Cognitive status was assessed using standard neuropsychological testing coupled with central adjudication by a panel of expert clinicians. DII Dietary Inflammatory Index, WHIMS Women’s Health Initiative Memory Study.

A total of 1528 women (baseline age: 71.3 [SD 3.8] years) without cognitive impairment at baseline were included in the analyses (Fig. 1). Distributions of baseline age, recruitment region, education level and obesity were unequal across low and high EDII groups (median split; Table 1). EDII scores in this study (range: −6.1 to 4.8; mean: −1.2; SD: 2.0) were comparable with those reported in earlier studies [27–30]. The average follow-up time (time to onset of CI or final follow-up visit) was 13.6 years (median: 14.4 IQR: 11.2, 18.1). Among women who developed CI at follow-up (*n* = 812), 462 (56.9%) met the criteria for MCI and 350 (43.1%) met the criteria for dementia.

### Inflammatory diet is associated with inflammatory protein expression

**Fig. 2:**
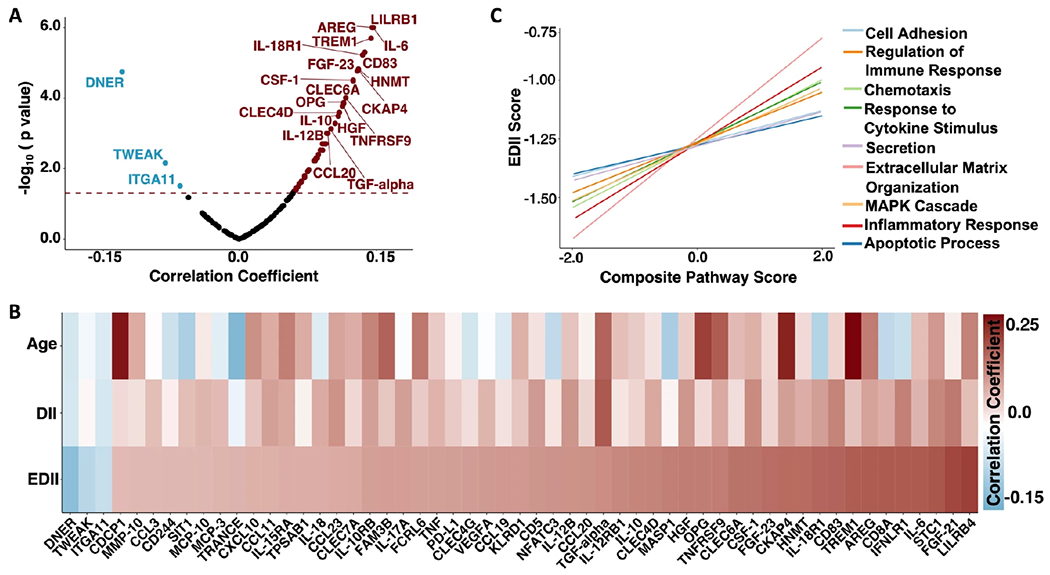
Association of inflammatory/immune proteins and pathways with Energy-Adjusted Dietary Inflammatory Index (EDII). **A** Volcano plot showing correlations between EDII and 151 individual protein levels. Proteins above the dashed horizontal line were statistically significant (*p* < 0.05). **B** Heatmap showing 55 individual proteins significantly correlated with EDII, as well as corresponding associations with Age and unadjusted DII scores. **C** Lines of best fit illustrating significant correlations between EDII and immune/inflammatory pathway composite scores. Results derived from partial Spearman correlations adjusted for age. DII Dietary Inflammatory Index, EDII Energy adjusted Dietary Inflammatory Index.

Among the 151 inflammatory/immune proteins included in analyses, 55 were significantly correlated with EDII scores after adjusting for age, including those select measures (IL-6, TNF) previously associated with an inflammatory diet (Fig. 2A, B; Supplementary Table 5). The strongest positive correlations were observed among proteins involved in MHC class I mediated antigen processing (LILRB4), metabolism of T-cells (FGF-21), regulation of phagocytosis (STC1), and pro-inflammatory cytokine signaling (IL-6). Three proteins were negatively correlated with EDII scores (DNER, TWEAK, ITGA11). The magnitude of the statistically significant age-adjusted associations between inflammatory/immune proteins and EDII scores was generally modest; Spearman correlations ranged from −0.11 to 0.15. The associations of 41 proteins with EDII scores were statistically significant following FDR correction.

Using Gene Ontology annotations, we then grouped 91 of the 151 inflammatory/immune proteins into 11 immunologically relevant biological pathways (average annotations per protein = 2.3 pathways), and for each pathway created a protein-based composite score. Nine of the 11 pathways maintained significant positive correlations with EDII after adjusting for age (Fig. 2C; Supplementary Table 6). EDII was most strongly associated with levels of the *extracellular matrix organization* (*rho* = 0.12; *p* < 0.001), *inflammatory response (rho* = 0.11; *p* < 0.001), and *response to cytokine stimulus* pathways (*rho* = 0.08; *p* = 0.001). Similar to findings of individual proteins, effect sizes for the association between EDII and inflammatory/immune composite scores were modest (range: 0.05 to 0.12).

### Inflammatory diet proteins and pathways are associated with cognitive impairment

**Fig. 3:**
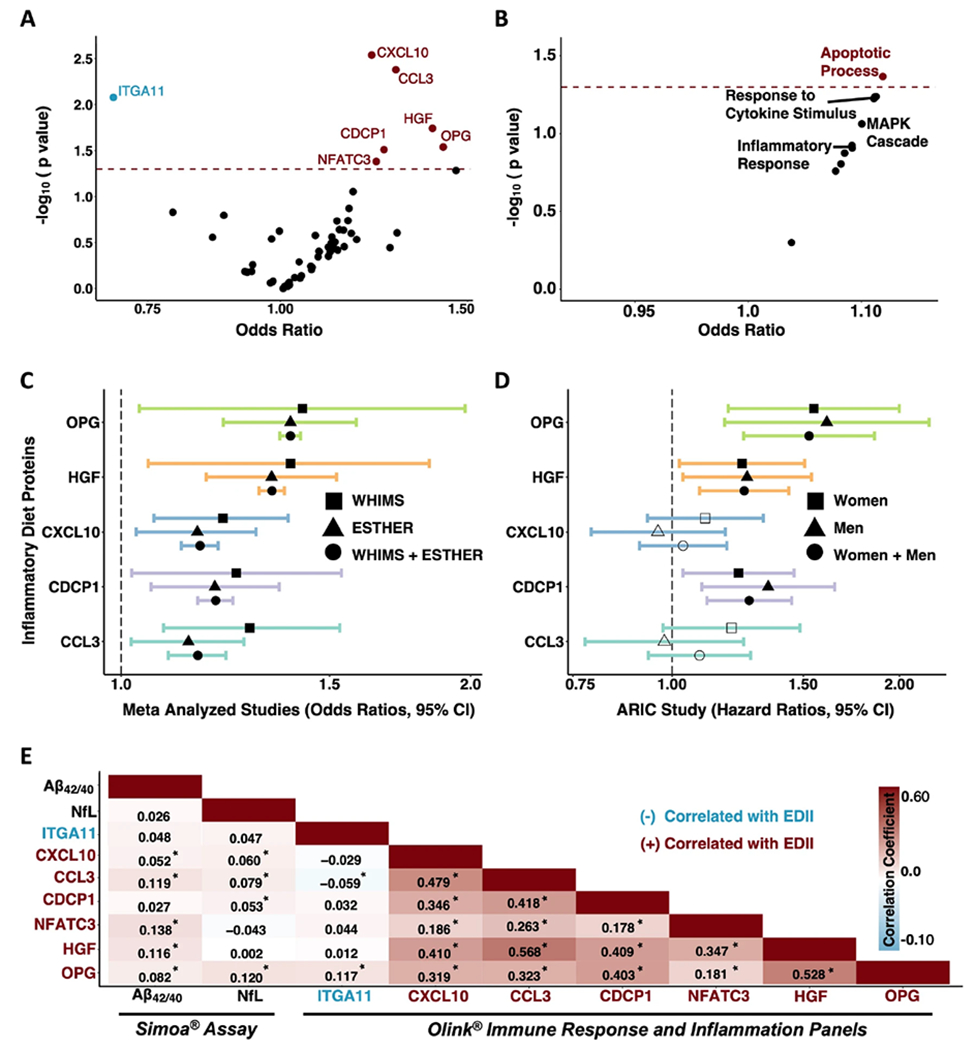
Inflammatory diet proteins and pathways related to incident cognitive impairment, validation of proteins in external cohorts and associations with neurodegenerative disease biomarkers. Volcano plots showing the associations of (**A**) inflammatory diet proteins and (**B**) pathways with odds of incident cognitive impairment derived from multivariable logistic regression models. Proteins and pathways above the dashed horizontal line were statistically significant (*p* < 0.05). Models adjusted for age, *APOE* ε4 status, education, obesity, diabetes, hypertension, eGFR-creatinine, geographical region, hormone treatment. **C** Forest plot showing significant associations (shaded shapes, *p* < 0.05) of inflammatory diet proteins with risk for cognitive impairment (WHIMS) and dementia (ESTHER), and meta-analyzed results (WHIMS + ESTHER). Results derived from multivariable logistic regression models adjusted for covariates used in WHIMS (see above) and ESTHER (age, *APOE* ε4 status, education, BMI, diabetes, cardiovascular disease, depression, physical activity, sex). **D** Forest plot showing associations of inflammatory diet proteins with time to dementia onset in the ARIC Study. Results derived from Cox proportional hazards regression models adjusted for covariates in ARIC (age, center-race, sex, education, *APOE* ε4 status, diabetes, BMI, smoking status, hypertension, and eGFR-creatinine). Two plasma proteins associated with cognitive impairment in WHIMS (NFATC3, ITGA11) were not measured in ESTHER or ARIC. **A**-**D**: Hazards and Odds ratios represent differences in risk per each log2 increase in protein level. **E** Heatmap showing correlations and corresponding coefficients between candidate proteins and plasma biomarkers of brain amyloid-β (Aβ_42/40_) and neurodegeneration (NfL). *Correlation statistically significant (*p* < 0.05). Results derived from partial Spearman correlations adjusted for age. ARIC Atherosclerosis Risk in Communities, EDII Energy adjusted Dietary Inflammatory Index, ESTHER Epidemiological Study on the Chances of Cure, Early Detection and Optimized Therapy of Chronic Diseases in the Elderly Population.

Next, we determined whether EDII-related proteins were associated with odds of future cognitive impairment. Among the 52 inflammatory/immune proteins positively correlated with EDII score, six proteins (CXCL10, CCL3, HGF, OPG, CDCP1, NFATC3) were significantly associated with increased odds of incident cognitive impairment after adjusting for demographic characteristics and cardiovascular risk factors (Fig. 3A). Among the three inflammatory/immune proteins negatively correlated with EDII scores, one protein (ITGA11) was significantly associated with decreased odds of incident cognitive impairment in the same covariate-adjusted model. Results were similar across adjusted and unadjusted models, and in sensitivity analyses that additionally adjusted for lifestyle factors (Supplementary Table 7). Prior evidence indicates three of these candidate proteins (OPG, CDCP1, ITGA11) play a role in membrane-bound receptor complexes, another three can function as secreted cytokines (CXCL10, HGF, CCL3), and one (NFATC3) primarily functions as a transcription factor. Disease annotations suggested three of the proteins are linked to different types of cancer (CDCP1, CCL3, ITGA11), whereas another two are related to liver diseases (CXCL10, HGF). Biological processes, molecular functions, and diseases associated with each protein are provided in Supplementary Table 8. The associations of candidate proteins with risk for cognitive impairment were not statistically significant following FDR correction. Of the nine inflammatory/immune pathways linked to the EDII score, only the *apoptotic process* composite score was significantly associated with higher odds of future cognitive impairment after adjusting for demographic characteristics and cardiovascular risk factors (OR 1.12, 95% CI 1.00, 1.25, *p* = 0.04; Fig. 3B; Supplementary Table 9).

### Inflammatory diet proteins are related to incident dementia in external cohorts

We next sought to validate the observed associations with cognitive impairment among EDII-related proteins using results from two external cohorts. We used computed results published by the ESTHER cohort (*n* = 1782; baseline age 63.3 [SD 2.3] years; 54.2% women), which examined the relationship between baseline serum inflammatory proteins (measured using the same Olink Inflammation panel) and incident dementia over a 17-year follow-up (median: 16.3 IQR: 13.5, 17.0) [34]. As displayed in Fig. 3C, five of the seven candidate proteins were measured in ESTHER;all five proteins (CCL3, CXCL10, CDCP1, HGF, OPG) were significantly associated with dementia risk and the direction of the association was consistent with that of the current study. For each of these five proteins, we also meta-analyzed the results from the current study and the results published by the ESTHER cohort. Pooled results from fully adjusted analyses across WHIMS and ESTHER revealed that higher levels of each protein were significantly associated with elevated odds of cognitive impairment (Fig. 3C; Supplementary Table 10).

As part of an additional protein validation step, we determined whether our candidate proteins were associated with time-to-dementia onset over an eight-year follow-up period in the ARIC study (*n* = 4288; baseline age 75.2 [SD 5.0] years; 57.9% women). See Supplementary Table 11 for ARIC sample characteristics. The ARIC study measured five of the seven candidate proteins using the SomaScan platform. Correlations of measurements for the same protein across Somascan and Olink platforms were calculated previously in ARIC and in the Fenland study [54]. With one exception (CCL3), cross-platform correlations for candidate proteins were strong (Supplementary Table 12). In the full ARIC sample, three of the five proteins (CDCP1, HGF, OPG) were significantly associated with incident dementia and the direction of the associations was consistent with that of the current study (Fig. 3D; Supplementary Table 13).

### Inflammatory diet proteins are associated with amyloid-β and neurodegeneration

To understand how inflammatory diet proteins may relate to the neurobiology underlying cognitive decline, we related the inflammatory/immune proteins and pathways associated with both EDII and incident cognitive impairment to plasma biomarkers of AD pathology and neurodegeneration measured at the same baseline visit. Four of the seven candidate proteins (CXCL10, CCL3, CDCP1, OPG) demonstrated significant positive correlations with plasma NfL, a marker of neuronal injury, after adjusting for age (Fig. 3E). Five of the seven candidate proteins were positively associated with Aβ_42/40_ ratio, suggesting that higher levels of these inflammatory diet proteins were associated with *lower* levels of brain amyloid (Fig. 3E; Supplementary Table 14). Similarly, the *apoptotic process* composite score, which was associated with a greater EDII score and incident cognitive impairment, also maintained a significant positive correlation with NfL (*rho* = 0.10, *p* < 0.001) and Aβ_42/40_ (*rho* = 0.19, *p* < 0.001) after adjusting for age (Supplementary Table 14).

### Inflammatory diet proteins relate to brain atrophy

**Fig. 4:**
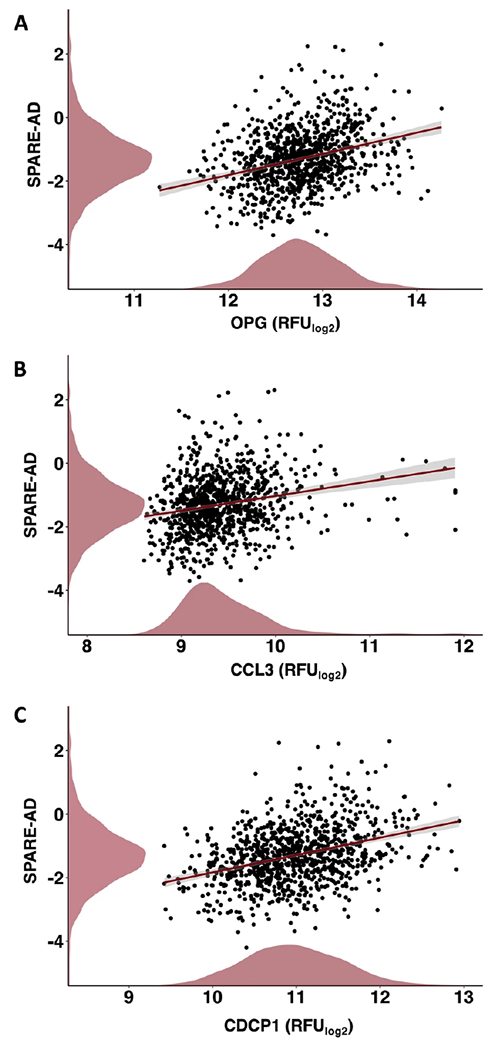
Associations of inflammatory diet proteins with the SPARE-AD, an MRI-defined index of neurodegenerative brain atrophy. Scatterplots and lines of best fit show levels of inflammatory diet proteins identified in WHIMS, **A** OPG, **B** CCL3, **C** CDCP1, significantly related to SPARE-AD scores in BLSA participants, as evidenced by multivariable linear regression models adjusted for age, sex, race, education, *APOE*ε4, eGFR-creatinine and a comorbidity index. Density plots along x-and y-axes display distributions of inflammatory diet proteins and SPARE-AD scores, respectively. CCL3 C-C motif chemokine 3, CDCP1 CUB Domain Containing Protein 1, OPG Osteoprotegerin, RFU Relative Fluorescence Intensities, SPARE-AD Spatial Pattern of Atrophy for Recognition of Alzheimer’s Disease.

We used data from the BLSA (*n* = 970;baseline age 66.0 [SD 14.8] years; 55.1% women) to understand how inflammatory diet proteins relate to differences in SPARE-AD, a brain MRI index that reflects multi-variate changes in brain structure that are observed in neurodegenerative phenotypes, particularly AD. For BLSA sample characteristics, see Supplementary Table 15. In analyses that were adjusted for demographic factors and comorbid conditions, three of the six candidate inflammatory diet proteins (OPG, CCL3, and CDCP1) related positively to SPARE-AD score (Fig. 4A–C), indicating that higher levels of each protein are associated with a greater atrophy in brain regions particularly vulnerable to AD-related neurodegeneration. Full results are provided in Supplementary Table 16.

### Characterization of inflammatory diet proteins linked to incident cognitive impairment

**Fig. 5:**
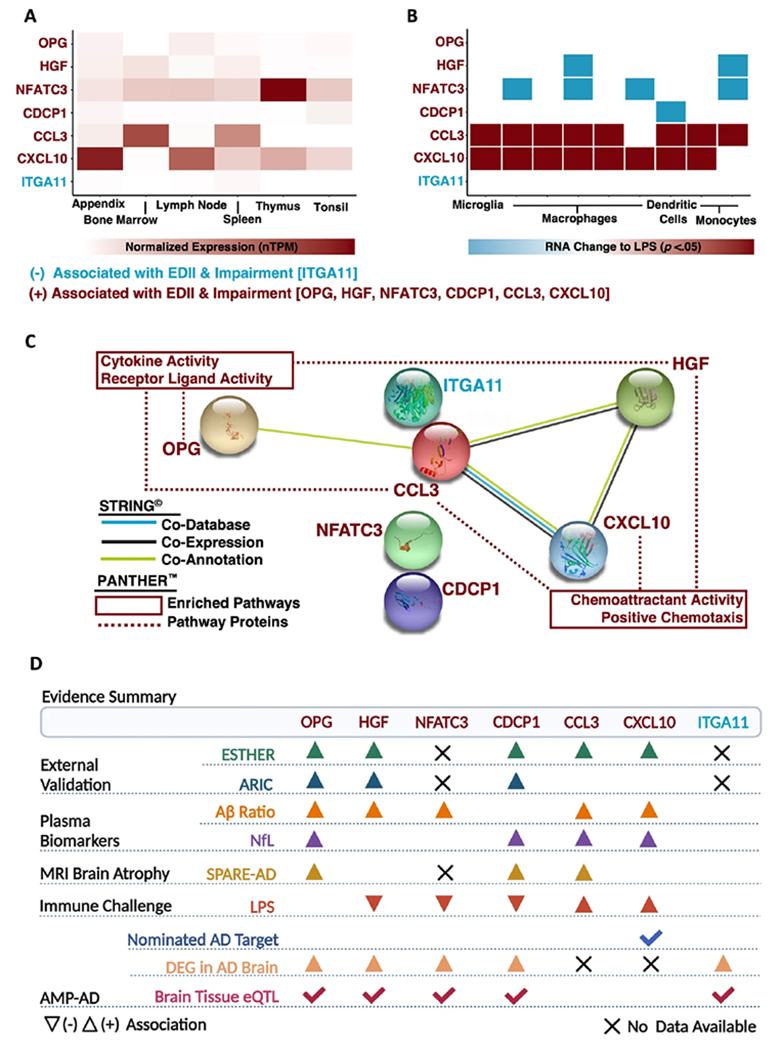
Characterization of inflammatory diet proteins linked to incident cognitive impairment. **A** Consensus transcript expression levels (normalized Transcripts per Million; nTPM) across immune tissues of interest based on transcriptomics data from the Human Protein Atlas and Genotype-Tissue Expression project. **B** Results from nine different RNA-seq analyses across four different immune cell types which reported differential expression of genes encoding candidate proteins in response to treatment with lipopolysaccharide (LPS), an immune challenge. **C** Protein-protein interaction network analyses were generated using STRING^©^; predicted protein conformations are depicted in each protein’s circular node. GO enrichment analysis for biological processes and molecular functions utilized PANTHER^™^ functional classification platform. **D** Summary of evidence, including results linking candidate proteins to data obtained from AMP^®^-AD. Accelerating Medicines Partnership for Alzheimer’s Disease AMP^®^-AD, eQTL expression quantitative trait loci, DEG differentially expressed gene, LPS lipopolysaccharide; normalized Transcripts per Million, nTPM Protein Analysis Through Evolutionary Relationships Classification System, PANTHER^™^ Search Tool for the Retrieval of Interacting Genes/Proteins, STRING^©^.

Finally, we sought to better understand the biological implications and functional relevance of the seven plasma proteins linked to EDII as well as cognitive impairment. First, we leveraged consensus transcript expression levels available through the Human Protein Atlas to examine the expression of genes encoding candidate proteins (CXCL10, CCL3, HGF, OPG, CDCP1, NFATC3, ITGA11) across specific immune tissues and cell types. *NFATC3* and *CXCL10* exhibited the highest expression levels in four of the six immune tissues of interest (appendix, lymph node, thymus, tonsil), whereas expression of *CCL3* was highest in two tissues (bone marrow, spleen). (Fig. 5A; Supplementary Table 17). Curated data from scRNA-seq analyses indicated B-cells, T-cells, and dendritic cells maintained the highest expression of *CCL3*, whereas granulocytes, monocytes, and natural killer cells illustrated the highest expression of *NFATC3* (Supplementary Table 17).

We also examined how the expression of genes encoding candidate proteins might be altered in response to an immune challenge. To assess this outcome, we utilized results from nine published studies that employed RNA-seq in conjunction with in vitro immune cells treated with LPS [50]. We observed a consistent upregulation of cytokines (*CXCL10, CCL3*) following immune challenge across cell types, including microglia. Conversely, findings indicated that LPS treatment induced downregulation of *NFATC3* and/or *HGF* in macrophages and monocytes, while downregulation of *CDCP1* was reported in dendritic cells (Fig. 5B; Supplementary Table 18). Such in vitro patterns of expression following an immune challenge indicate the positive associations of inflammatory diet with candidate proteins in the current study may reflect pro-inflammatory responses (CXCL10, CCL3) or compensatory, anti-inflammatory patterns of regulation (NFATC3, HGF, CDCP1) that alter risk for subsequent cognitive impairment.

To further understand candidate protein biology and determine their potential role in AD, we utilized several complementary, publicly available resources. Using STRING protein-protein interaction network analyses and functional enrichment, we found that three of our candidate proteins (CXCL10, CCL3, HGF) involved in cellular locomotion in response to chemical stimulus (i.e., chemoattractant activity, positive chemotaxis) also exhibited co-expression patterns (Fig. 5C; Supplementary Tables 19, 20). Leveraging data obtained from comprehensive post-mortem brain tissue collections, along with expression data via the AMP-AD Sage Bionetworks Agora platform, we also observed five of our candidate proteins (OPG, HGF, NFATC3, CDCP1, ITGA11) display upregulated expression in the brains of AD individuals and maintain expression quantitative trait loci (eQTL) in brain tissue. These results suggest that at least five of the seven inflammatory diet proteins identified in plasma and linked to cognitive impairment may be differentially expressed in the brains of individuals at risk for AD and regulated, at least in part, by underlying genetic variation (Fig. 5D). While one of the proteins linked to EDII and incident cognitive impairment (CXCL10) has been nominated as a therapeutic target for AD, three candidate proteins (CCL2, CXCL10, and HGF) are therapeutic targets of ongoing clinical trials for non-neurologic disease (Supplementary Table 21).

## DISCUSSION

The current findings provide insights into how inflammatory nutritional patterns relate to an immune-related plasma proteome. In addition to identifying a set of inflammatory diet proteins whose levels were associated with risk for cognitive impairment over a 14-year follow-up period, we demonstrated that several of these same proteins were associated with dementia risk in two external cohorts and correlated with plasma biomarkers of AD pathology (Aβ_42/40_) and/or neurodegeneration (NfL). Using 3T MRI data from a separate sample, we also found that several inflammatory diet proteins were related to an index of neurodegenerative brain atrophy. Along with evaluating their biological relevance, assessing their potential role in AD, and characterizing their immune-tissue/cell-specific expression, we report that in vitro regulation of genes encoding these candidate proteins might be altered in response to an immune challenge [50]. Together, these results highlight the molecular mediators through which an inflammatory diet may contribute to the risk for age-related cognitive impairment and dementia.

While previous investigations have demonstrated a relationship between inflammatory diet and a select group of inflammatory proteins (e.g., CRP, IL6, TNF), the current study extends these results by demonstrating that inflammatory diet is associated with a much larger segment of the immune proteome (55 proteins, adjusting for age) [5–8]. Several lines of evidence indicate that the patterns of protein expression reported here indeed reflect diets with differing inflammatory potential. First, we observed significant correlations of EDII scores with increasing levels of IL-6 and TNF, two pro-inflammatory cytokines previously used to validate anti-inflammatory diets (e.g., Medi Diet) and inflammatory diet indices [5, 7, 8, 55–57]. In addition, many of the proteins significantly correlated with energy-adjusted diet scores in our study (FGF-21, IL-6, IL-18R1, FGF-23, CSF-1, HGF, CCL20, IL-12B, VEGFA, IL-10RB, IL-18, TRANCE, CCL3, CDCP1, TWEAK) were also related to BMI in a recently published RCT, where changes in these proteins also tracked changes in BMI following a healthy dietary intervention [33]. The upregulation of nine immunologically relevant pathways in the context of higher EDII scores further supports the concept of a broad, diet-induced immune stimulation, and calls attention to specific cellular processes (*extracellular matrix organization, inflammatory response, response to cytokine stimulus*) by which pro-inflammatory diets may contribute to disrupted cellular homeostasis. Notably, the *apoptotic process* composite score, which was positively associated with baseline EDII scores as well as incident cognitive impairment in this study, was also found in the Swedish BioFINDER study to distinguish amyloid-positive AD and amyloid-positive MCI participants from amyloid-negative cognitively normal and MCI individuals [35]. Adding to these previous findings, our results suggest that the regulation of proteins in this cell-death signaling cascade may be augmented by an inflammatory diet and relate—perhaps mechanistically—to amyloid deposition, at least during the initial stages of cognitive decline.

The candidate proteins we identified have been associated with cognitive decline in other studies using similar proteomic assays, providing further support to our findings. Higher plasma levels of CDCP1, OPG, and HGF have been shown to accurately discriminate dementia cases from cognitively normal older adults [58–60]. Furthermore, differing concentrations of plasma ITGA11 and NFATC3, and elevated intrathecal CCL3 have been observed among individuals diagnosed with AD, while abnormal levels of CXCL10 in CSF or plasma are found across multiple neurodegenerative phenotypes, including FTD and AD [59, 61–63]. Of note, we observed consistency in directionality of the relationship between protein level and cognitive impairment, (i.e., proteins positively associated with an inflammatory diet were associated with *greater* cognitive impairment, and vice-versa). The consistency of these results reinforces prior studies that report increased dementia incidence tied to pro-inflammatory diets [11–15] and extends these findings by revealing specific plasma proteins that may link an inflammatory diet to cognitive dysfunction in aging.

Our assessment of inflammatory diet proteins in relation to plasma biomarkers of AD pathology and neurodegeneration adds to the limited understanding of how dietary patterns may influence pathological processes within the CNS [64–66]. Of the seven plasma proteins linked to pro-inflammatory diet and cognitive impairment, four and three were positively associated with NfL and an MRI measure of AD-relevant brain atrophy, respectively. These findings support the well-documented relationship between peripheral inflammation and neurodegeneration [67–70]. Perhaps more striking are our findings that inflammatory-diet proteins linked to cognitive impairment tend to be associated with lower brain Aβ levels (as defined by a greater plasma Aβ_42/40_ ratio). These results are consistent with recent data suggesting immune activation may limit deposition of brain Aβ among individuals at-risk for cognitive deficits [71–73]. While it is unlikely that an inflammatory diet has a protective effect on Aβ pathology, individuals predisposed to mount a more robust immune response to an inflammatory diet may also mount a stronger response to brain Aβ deposition, at least initially. While the observational nature of this study precludes any determination of causality, our results nonetheless suggest that proteins linked to an inflammatory diet may contribute to neuronal injury. We note, however, that although we show CCL3 and OPG are positively associated with neurodegeneration biomarkers in two separate cohorts, other investigations have failed to find associations between plasma CCL3, OPG, and atrophy in brain regions commonly implicated in AD [74, 75].

Using comprehensive transcriptomic datasets coupled with published in vitro RNA-seq analyses, we found that three candidate proteins, CCL3, CXCL10 and NFATC3, maintained the highest expression levels across immune tissues/cells and displayed consistent differential expression in response to LPS treatment. These three proteins may be especially responsive to immunologic stressors, and in the context of our results, may play a role in regulating the effects of pro-inflammatory processes on target cells within the brain. In addition to demonstrating their relationship to inflammatory diet and incident cognitive impairment/dementia, we identified OPG, HGF, NFATC3, CDCP1, and ITGA11 as differentially expressed at the RNA level in brain tissue of deceased individuals with pathologically defined AD. While all five proteins have been previously implicated in AD, our results suggest that an inflammatory diet may be a key driver of these AD-associated proteins [58–63]. Interestingly, three of the seven candidate proteins are cytokines with known patterns of coexpression. One of these proteins, CXCL10 (also known as interferon gamma-induced protein 10), acts as a ligand for CXCR3, thereby promoting activation of monocytes and natural killer cells, as well as the migration of T-cells [76]. In an amyloid precursor protein (APP)/presenilin 1 (PS1) AD mouse model, antagonism of the CXCL10 receptor resulted in increased microglial phagocytosis of Aβ, reduced neuroinflammation, and attenuated behavioral deficits [77]. Accordingly, CXCL10 has been nominated by the Accelerated Medicine Partnership as a potential therapeutic target for AD. We also note that CXCL10 is currently an experimental drug target for multiple autoimmune/inflammatory conditions, suggesting that the repurposing of these drugs to treat AD/ dementia should be considered, particularly among individuals at risk for chronic inflammation.

Although our study has several strengths, including the detailed collection of nutritional data, the application of state-of-the-art proteomics, a multi-cohort replication, the inclusion of AD/ neurodegenerative biomarkers, and the incorporation of 3 T MRI data from a separate cohort, our findings should be interpreted within the context of several limitations. First, because of the goals of the parent study (WHIMS ancillary study), participants were limited to white women. Although protein-dementia associations replicated in more diverse external samples, additional studies will be needed to determine the generalizability of results to other demographic groups. Second, the use of a matched case-control design for the WHIMS ancillary study precluded the use of time-to-event analyses, which may be better suited to detect protein-cognitive impairment associations. Third, while an inflammatory diet was previously associated with risk for incident cognitive impairment in a group of 7085 participants from the parent WHIMS study [14], the current analysis was restricted to a more deeply phenotyped subset of participants at approximately one-fifth the size. In turn, limited power to detect the DII-cognitive impairment association demonstrated previously precluded the use of formal mediation models. Fourth, given the observational nature of this study, the extent to which diet and proteins exert causal effects, as well as the directionality of these effects, are unknown. We encourage future preclinical investigations to establish temporal precedence and the associated causality between components of pro-inflammatory diets (e.g., saturated fats) and ensuing variation in the immune-related proteome. Fifth, the associations between inflammatory protein levels and inflammatory diet scores were weak, consistent with prior reports [29, 78–80]. This could suggest that diet only plays a limited role in determining one’s inflammatory milieu, particularly in the context of contributions from other factors (e.g., genetic variation, lifetime exposure to pathogens/infections, etc.) or that measurement error inherent in the retrospective self-reported dietary assessment [81] attenuated true associations between inflammatory diet scores and inflammatory protein levels. In the case of the latter, we believe there may be room for new tools which more accurately quantify an individual’s inflammatory diet. Additionally, biological fluctuations (e.g., diurnal and day-to-day variation) may lead to variation in protein measures that could have limited our ability to detect associations of proteins with inflammatory diet and cognitive impairment [82]. Therefore, null effects should be interpreted with appropriate caution. However, numerous studies have demonstrated that outside the context of an acute infection or injury, levels for many inflammatory proteins—and plasma proteins more broadly—remain relatively stable across time [83, 84]. Despite these limitations, the current results provide insights into the molecular conduits through which pro-inflammatory nutritional habits may contribute to cognitive impairment. Follow-up studies will be needed to determine whether the identified proteins indeed play a causal, mechanistic role in late-life cognitive decline and dementia.

## Supplementary Material

Supplementary Materials

## Figures and Tables

**Table 1.** Baseline sample characteristics from the Women’s Health Initiative Memory Study.

		Total Sample (*n* = 1528)	Low EDII (*n* = 764)	High EDII (*n* = 764)
Demographics	EDII^a^	−1.22 (1.97)	−2.85 (0.98)	0.41 (1.24)
	DII^a^	1.25 (2.32)	0.64 (2.37)	1.86 (2.11)
	Age^a^	71.30 (3.75)	71.67 (3.72)	70.93 (3.74)
	Female	1528 (100.0%)	764 (100.0%)	764 (100.0%)
	white	1528 (100.0%)	764 (100.0%)	764 (100.0%)
Region	Northeast	457 (29.9%)	221 (28.9%)	236 (30.9%)
	South	329 (21.5%)	181 (23.7%)	148 (19.4%)
	Midwest^a^	356 (23.3%)	152 (19.9%)	204 (26.7%)
	West	386 (25.3%)	210 (27.5%)	176 (23.0%)
Treatment Assignment	Placebo	744 (48.7%)	377 (49.3%)	367 (48.0%)
	Estrogen	784 (51.3%)	387 (50.7%)	397 (52.0%)
Education	Less than high school	78 (5.1%)	32 (4.2%)	46 (6.0%)
	High school or GED^a^	312 (20.5%)	128 (16.8%)	184 (24.2%)
	Some college	600 (39.4%)	308 (40.4%)	292 (38.4%)
	Graduated college^a^	534 (35.0%)	295 (38.7%)	239 (31.4%)
*APOE*ε4 Alleles	0 ε4 alleles	894 (58.5%)	441 (57.7%)	453 (59.3%)
	1-2 ε4 alleles	634 (41.5%)	323 (42.3%)	311 (40.7%)
Cardiovascular Risk Factors	Hypertension	729 (47.7%)	353 (46.2%)	376 (49.2%)
	Diabetes	101 (6.6%)	55 (7.2%)	46 (6.0%)
	Obesity^a^	499 (32.8%)	203 (26.6%)	296 (39.1%)
Kidney Function	eGFR	79.99 (12.82)	80.05 (12.30)	79.92 (13.33)
Follow-Up Time	Years	13.55 (5.20)	13.40 (5.23)	13.69 (5.17)
Cognitive Status (At Final Follow-Up)	Cognitively Unimpaired	716 (46.9%)	359 (47.0%)	357 (46.7%)
	Cognitively Impaired	812 (53.1%)	405 (53.0%)	407 (53.3%)

Values are displayed as means (standard deviation) and frequencies (percentages). Median split was used to identify Low/High EDII. We utilized *t*-tests for continuous variables and χ^2^ for categorical variables.

*DII* Dietary Inflammatory Index, *EDII* Energy Adjusted Dietary Inflammatory Index, *eGFR* estimated glomerular filtration rate (eGFR)-creatinine.

a Difference between low DII and high DII groups statistically significant (*p* < 0.05).
