# Correction to: Plasma proteins related to inflammatory diet predict future cognitive impairment

**DOI:** 10.1038/s41380-023-02007-0

**Published:** 2023-02-27

**Authors:** Michael R. Duggan, Lauren Butler, Zhongsheng Peng, Gulzar N. Daya, Abhay Moghekar, Yang An, Stephen R. Rapp, Kathleen M. Hayden, Aladdin H. Shadyab, Ginny Natale, Longjian Liu, Linda Snetselaar, Ruin Moaddel, Casey M. Rebholz, Kevin Sullivan, Christie M. Ballantyne, Susan M. Resnick, Luigi Ferrucci, Keenan A. Walker

**Affiliations:** 1 Laboratory of Behavioral Neuroscience, National Institute on Aging, Baltimore, MD USA; 2 Department of Neurology, Johns Hopkins University School of Medicine, Baltimore, MD USA; 3 Department of Social Sciences and Health Policy, Wake Forest University School of Medicine, Winston-Salem, NC USA; 4 Department of Psychiatry & Behavioral Medicine, Wake Forest University School of Medicine, Winston-Salem, NC USA; 5 Herbert Wertheim School of Public Health and Human Longevity Science, University of California, San Diego, La Jolla, CA USA; 6 Program in Public Health, Stony Brook University School of Medicine, Stony Brook, NY USA; 7 Department of Epidemiology and Biostatistics, Drexel University Dornsife School of Public Health, Philadelphia, PA USA; 8 Department of Epidemiology, University of Iowa, Iowa City, IA USA; 9 Laboratory of Clinical Investigation, National Institute on Aging, Baltimore, MD USA; 10 Department of Epidemiology, Johns Hopkins Bloomberg School of Public Health, Baltimore, MD USA; 11 Department of Medicine, University of Mississippi Medical Center, Jackson, MS USA; 12 Section of Cardiovascular Research, Department of Medicine, Baylor College of Medicine, Houston, TX USA; 13 Translational Gerontology Branch, National Institute on Aging, Baltimore, MD USA

**Keywords:** Prognostic markers, Neuroscience

Correction to: *Molecular Psychiatry* 10.1038/s41380-023-01975-7, published online 03 February 2023

The article “Plasma proteins related to inflammatory diet predict future cognitive impairment”, written by Michael R. Duggan, Lauren Butler, Zhongsheng Peng, Gulzar N. Daya, Abhay Moghekar, Yang An, Stephen R. Rapp, Kathleen M. Hayden, Aladdin H. Shadyab, Ginny Natale, Longjian Liu, Linda Snetselaar, Ruin Moaddel, Casey M. Rebholz, Kevin Sullivan, Christie M. Ballantyne, Susan M. Resnick, Luigi Ferrucci & Keenan A. Walker, was originally published electronically on the publisher’s internet portal on 3 February 2023 without open access. With the author(s)’ decision to opt for Open Choice the copyright of the article changed on 10 February 2023 to © The Author(s) 2023 and the article is forthwith distributed under a Creative Commons Attribution 4.0 International License, which permits use, sharing, adaptation, distribution and reproduction in any medium or format, as long as you give appropriate credit to the original author(s) and the source, provide a link to the Creative Commons licence, and indicate if changes were made. The images or other third party material in this article are included in the article’s Creative Commons licence, unless indicated otherwise in a credit line to the material. If material is not included in the article’s Creative Commons licence and your intended use is not permitted by statutory regulation or exceeds the permitted use, you will need to obtain permission directly from the copyright holder. To view a copy of this licence, visit http://creativecommons.org/licenses/by/4.0.

