# Supplementary material for: Plasma proteins related to inflammatory diet predict future cognitive impairment
Source: Mol Psychiatry. Author manuscript; Available in PMC 2023 May 27. (PMC10208977; doi:10.1038/s41380-023-01975-7)
Supplement: Supplementary Materials [file NIHMS1890179-supplement-Supplementary_Materials.pdf]

SUPPLEMENTARY METHODS

Study Sample

Women's Health Initiative Memory Study (WHIMS) participants were recruited from 39 sites of the larger Women's Health Initiative (WHI) study (enrollment: May 1996- December 99). Details of the study design and the initial screening process have been published elsewhere in full[1-5]. Written informed consents were obtained from all participants. The study design, data collection, and analyses in this study were approved by the institutional review boards of WHIMS participating centers. WHIMS was initially comprised of randomized clinical trials for the evaluation of the effects of estrogen alone (E-Alone) or in combination with progestin (E+P) on incident MCI or probable dementia [6, 7]. The WHI E+P trial and the E-Alone trial ended in July 2002 and February 2004, respectively [1-4, 8]. Participants continued post-trial cognitive assessments, returning for annual clinic visits to evaluate cognitive function until July 2008 at the latest, when the study transitioned to the WHIMS–Epidemiology of Cognitive Health Outcomes (NCT00745056), a study where annual cognitive assessments were conducted by telephone. At baseline enrollment in WHI, blood samples were collected and stored for later analysis using standard protocols, and participants completed the WHI Food Frequency Questionnaire (WHI-FFQ; based on the Block FFQ)[6, 9-12]. Participants were eligible for inclusion in the present analysis if they completed more than one follow-up cognitive examination, did not have cognitive impairment at baseline, provided WHI-FFQ responses at baseline and had available proteomic data from a WHIMS ancillary study that passed Olink quality control (QC).

The WHIMS ancillary study, called WHIMS Resiliency, was designed to achieve a better understanding of factors that protect against the development of clinical symptoms of Alzheimer's Disease (AD), even in the presence of the *APOE ε4* risk allele. Using a matched case-control design, the sampling strategy and participant selection were based on *APOE* genotype (presence of *ε4* risk allele), cognitive status (cognitively unimpaired, cognitively impaired), and age (≤ 80 vs >80). Sixty women with ε2/ε4 alleles were excluded due to opposing effects of *ε2* and *ε4* alleles on amyloid accumulation and longevity, and the sample was limited to Caucasian women due to different *APOE* genotype frequencies in African Americans.

Energy-Adjusted Dietary Inflammatory Index (EDII)

The Dietary Inflammatory Index (DII) was developed by Shivappa and colleagues at the University of South Carolina based on evidence suggesting that dietary factors influence inflammation. It characterizes an individual's diet on a continuum from maximally anti-inflammatory to maximally pro-inflammatory, with a higher DII score indicating a more pro-inflammatory diet and a lower DII score indicating a more anti-inflammatory diet[13]. A description of the DII and its construct validation have been reported previously[13-18]. Instead of being a specific food-based index, the DII represents a mixture of eight pro-inflammatory nutrients, 19 anti-inflammatory nutrients, 10 whole foods and spices, caffeine, flavan-3-ol, flavones, flavonols, flavanones, anthocyanidins, and isoflavones. In brief, its development and validation involved an extensive review of the literature, whereby 45 food items and nutrients were found to be associated with six well-known inflammatory biomarkers (IL-1β, IL-4, IL-6, IL-10, TNF-α, and CRP)[13]. The strength of the associations between the dietary factors and these inflammatory biomarkers reported in the literature were then scored: +1, 0, or −1 for a positive, null, or inverse association, respectively. Scores were then weighted by the type of study design supporting the associations. Weighted scores were tallied to obtain component-specific inflammatory effect scores. Dietary intake data were standardized to a representative global diet database that was constructed based on 11 data sets representing diverse populations around the world. For this analysis, 32 food parameters were available from the WHI-FFQ[19]. The WHI-FFQ is based on a modified Block FFQ that estimated mean daily nutrient intake during the previous 3-month period and includes 122 composite

and single-food line items asking about frequency of consumption and portion size, 19 adjustment questions related to the type of fat intake, and 4 summary questions about the usual intakes of fruits and vegetables and fats added in cooking or at the table[9, 10]. The standardized dietary intake estimates of each participant were converted to centered percentiles for each DII component, to improve interpretability; centered percentiles were then multiplied by the corresponding DII component-specific inflammatory effect scores and summed to obtain the overall DII score for each individual. Scores were then adjusted for energy consumed per 1,000 kcal (EDII) based on total energy intake derived from the WHI-FFQ[20-23].

Cognitive Assessment

In the first phase of WHIMS (1996-99 through 2007-2008), women received in-person examinations, as described previously[7]. Briefly, a modified version of the Mini–Mental State Examination (Modified Mini–Mental State Examination [3MS]) was administered annually to all participants[24]. Women scoring below preset cut-points based on education were referred for further evaluation including a clinical evaluation by a board-certified physician and additional neuropsychological testing including portions of the Consortium to Establish a Registry for Alzheimer's Disease battery[25], the Mini– Mental State Examination[26], Trail Making Test Parts A and B [27], a structured psychiatric interview (PRIME-MD)[28], the 15-item Geriatric Depression Scale-short form[29, 30], and a knowledgeable family member or friend was interviewed regarding cognitive and behavioral changes[7]. All data were centrally adjudicated by a panel of expert clinicians who classified each participant as MCI [31] or probable dementia[32] (Cognitively Impaired; CI) or no impairment (Cognitively Unimpaired; CU). In the extension phase of WHIMS (2007-2008 through 2018), the study transitioned to a telephone follow-up interview and cognitive assessment with the modified Telephone Interview for Cognitive Status (TICS-m)[33], the Oral Trail Making Test parts A and B[34], East Boston Memory Test[35], Digit Span[36], and Verbal Fluency-Animals[37]. Functional status was assessed with the Dementia Questionnaire (DQ)[38]. The DQ was administered to a proxy informant when TICS-m scores fell below 31 points. Similar to the first phase of WHIMS, all data in the extension phase were then used to adjudicate MCI or probable dementia (Cognitively Impaired; CI) or no impairment (Cognitively Unimpaired; CU).

Additional Covariate Measurement

Sleep disturbance was measured using Women's Health Initiative Insomnia Rating Scale (WHIMRS) [39], which is a measure of perceived insomnia symptoms and consists of five questions that assess insomnia and sleep quality during the past four weeks: "Did you have trouble falling asleep?"; "Did you wake up several times at night?"; "Did you wake up earlier than you planned to?"; "Did you have trouble getting back to sleep after you woke up too early?"; and "Overall, was your typical night's sleep during the past 4 weeks: Very sound or restful, sound or restful, average quality, restless, or very restless?" Response categories ranged from 0 ("No, not in the past 4 weeks") to 4 ("Yes, 5 or more times a week"), with a summed sleep quality score of the 5 items ranging from 0 to 20. Physical activity was measured as self-reported episodes per week of moderate and strenuous recreational physical activity, including walking fairly fast or very fast, moderate physical activity and strenuous physical activity. Smoking status was measured as self-reported never, past, or current smoker, while alcohol intake was measured as self-reported non-drinker, former, less than or equal to 7 drinks/week, or more than 7 drinks/week.

Atherosclerosis Risk in Communities (ARIC) Neurocognitive Study

A detailed description of ARIC and the ARIC proteomic measurements have been detailed previously [40]. The ARIC-Neurocognitive Study is an ongoing community-based study that initially enrolled 15,792 adults ages 45-65 between 1987 and 1989[41]. Participants were recruited from four sites across the United States: Washington County, MD; Forsyth County, MC; northwestern suburbs of Minneapolis, MN;

and Jackson, MS. Participants had three additional in-person visits, after which they were invited back for their fifth visit (visit 5; 2011-2013). At visit 5, blood was drawn for proteomic analysis. Participants were invited back for visit 6 (2016-2017) after five years, and visit 7 (2018-2019) immediately thereafter. Study protocols were approved by institutional review boards at each participating center: University of North Carolina at Chapel Hill, Chapel Hill, NC; Wake Forest University, Winston-Salem, NC; Johns Hopkins University, Baltimore, MD; University of Minnesota, Minneapolis, MN; and University of Mississippi Medical Center, Jackson, MS. All ARIC participants gave written informed consent at each study visit; proxies provided consent for participants who were judged to lack capacity.

Proteins were measured using the SOMAmer-based capture array method (SomaScan platform) [42] using plasma from blood samples collected at ARIC visit 5 using standardized protocols and frozen at -80°C. The SomaScan platform uses modified aptamers – short single-stranded DNA with chemically modified nucleotides - as protein-binding reagents which are identifiable and quantifiable using DNA detection technology. SomaLogic and ARIC protein quality control steps have been described in detail previously [40]. Using a set of 197 blind duplicates, intra-assay CVs were calculated. The 5 proteins included in this analysis had CVs <5%. Protein levels were log2 transformed to correct for skewness.

Dementia was classified between ARIC visits 5 and 7 using a surveillance approach that has been described in detail previously[40]. During visits 5, 6, and 7, participants received a comprehensive cognitive exam and a functional assessment that included the Clinical Dementia Rating Scale (CDR) and Functional Activities Questionnaire (FAQ). Using this data, dementia was classified based on the NIA/AA (National Institute on Aging and Alzheimer's Association) and the Diagnostic and Statistical Manual of Mental Disorder – Fifth Edition (DSM-5) criteria [43, 44]. In the time between visit 5 and visit 6 participants were contacted annually via phone and administered the SIS, a brief cognitive assessment[45]. If participants received a low score on the SIS, or if they were unable to participate in the screening via phone, the Ascertain Dementia 8 (AD8)[46] was administered to the participant's informant. For participants who received a dementia diagnosis at visit 6 or 7, SIS, AD8, hospital discharge, and death certificate codes were used to define the date of dementia onset. For participants who did not attend visits 6 or 7, SIS, AD8, hospital discharge, and death certificate codes were used to define dementia diagnosis and date of dementia onset.

Baltimore Longitudinal Study of Aging

The Baltimore Longitudinal Study of Aging (BLSA) is an ongoing longitudinal study in Baltimore, MD designed to assess physical and cognitive measures in a cohort of community-dwelling volunteers[47]. The BLSA protocol was approved by the Institutional Review Board of the National Institute of Environmental Health Science, National Institutes of Health; informed consent was obtained from all participants. A detailed description of the BLSA study design and procedures has been published elsewhere[47].

Regional brain volume measurements from 3T magnetic resonance imaging (MRI) were obtained as previously described[48-50]. MRI scan collection was initiated between 2009-2010. T1-weighted magnetization-prepared rapid gradient echo (MPRAGE) scans were acquired on a 3-T Philips Achieva or Ingenia (repetition time [TR] = 6.8 ms, echo time [TE] = 3.2 ms, flip angle = 8°, image matrix = 256 × 256, 170 slices, pixel size = 1 × 1 mm, slice thickness = 1.2 mm). By combining different atlases, warping algorithms, standardizing parameters, and using a consensus labeling approach to fuse these labels into a final segmentation, the Multi-atlas Region Segmentation Utilizing Ensembles (MUSE) anatomic labeling method generates an ensemble of labeled atlases in target image space [51]. We calculated voxel-wise tissue density maps for different brain tissue types using the RAVENS methodology (Regional Analysis of Volumes Examined in Normalized Space)[52]. To achieve optimal consistency between regional (volumetric) and voxel-wise analyses, we used MUSE ROI labels to segment the brain into GM, WM, VN, and CSF for RAVENS map calculations. RAVENS map intensity values quantify the

regional tissue densities for a subject at each voxel on a common template space, with one RAVENS map for each tissue type. RAVENS maps allow calculating voxel-wise statistical maps from a group of subjects without using prior regional definitions. The RAVENS approach has been extensively used and validated in large-scale neuroimaging studies[53, 54]. Using these estimates, we calculated a machine learning-based score known as the SPARE-AD (Spatial Pattern of Atrophy for Recognition of Alzheimer's Disease) which captures multi-variate changes in brain structure that accurately discriminate cognitively normal subjects from other neurodegenerative phenotypes, particularly AD[55-58]. In brief, it is computed by training a support vector machine (SVM) classification model to distinguish cognitively normal from clinically diagnosed dementia populations using structural brain features and has been shown to discriminate between normal cognition and mild cognitive impairment (MCI) as well as conversion from MCI to dementia [59-62].

Proteins were measured using the SOMAmer-based capture array method (SomaScan platform) [42], similar to the description above. Plasma samples were collected at the same visit as the MRI visit that was used to capture SPARE-AD, and, for a subset of participants, at the time of a first PET scan as part of a separate study; collection used standardized protocols and samples were frozen at -80°C. Samples were excluded if they did not pass SomaLogic QC criteria. Using a set of 102 blind duplicates, intra-assay CVs were calculated. The 6 proteins included in this analysis each had CVs ≤ 5%. Protein values were log2 transformed and those beyond 5 SDs from the mean were winzorized.

Protein Characterization

Gene Ontology (GO) annotations were obtained for Biological Processes and Molecular Functions from GeneCards (https://www.genecards.org). Disease annotations were obtained from Malacards (https://www.malacards.org). Supplemental information relevant to Alzheimer's Disease was obtained from the AD Knowledge Portal (https://adknowledgeportal.synapse.org), a platform for accessing data, analyses, and tools generated by the Accelerating Medicines Partnership (AMP-AD) Program for Alzheimer's Disease and other NIA-supported programs. The Therapeutic Target Database (http://db.idrblab.net/ttd/) was used to identify ongoing clinical trials that target specific proteins. GO enrichment analysis utilized PANTHER (Protein Analysis Through Evolutionary Relationships) functional classification platform (http://www.pantherdb.org). Protein interaction networks were assessed using STRING (Search Tool for the Retrieval of Interacting Genes/Proteins) (https://string-db.org), a database and visualization tool that integrates publicly available sources of information to provide a comprehensive understanding of protein–protein interaction networks.

Statistical Analysis

We used partial Spearman correlations (adjusted for age) to relate proteomic variables (individual proteins, pathway composite scores) to EDII scores, DII scores, and age. Subsequently, multivariable logistic regression models were used to examine associations of EDII-related proteomic variables with odds of cognitive impairment, adjusting for each of the demographic, physiological, and cardiovascular risk factors listed in the Covariate section in the main body of the manuscript (primary model). Unadjusted models were also examined. We also conducted sensitivity analyses to examine the effect of adjusting for lifestyle factors, which included smoking status, alcohol use, physical functioning and sleep quality. Next, from the ESTHER cohort, we extracted results for candidate inflammatory proteins that were related to 17-year dementia risk[63] . We pooled the ESTHER and WHIMS cohort-specific effect estimates using a fixed-effect, inverse variance-weighted meta-analysis to enhance the accuracy and generalizability of our results[64, 65]. We leveraged data from another external cohort (ARIC) to validate the association between individual proteins and time to cognitive impairment diagnosis. First, we examined the correlation between SomaScan and Olink measurement of the same candidate protein using Spearman and Pearson correlations. Candidate immune/inflammatory proteins measured in ARIC were

then examined in relation to eight-year dementia risk using Cox proportional hazards regression models adjusted for age, sex, race-center, education, *APOE*ε4, eGFR-creatinine, and cardiovascular risk factors (BMI, diabetes, hypertension, and current smoking status). To determine whether candidate proteins are associated with plasma biomarkers of AD pathology ($A\beta_{42/40}$) and neurodegeneration (NfL), we calculated partial Spearman correlations (adjusted for age). To assess how candidate proteins measured in BLSA relate to an index of neurodegenerative brain atrophy, specifically SPARE-AD, we used multivariable linear regression models adjusted for age, sex, race, education, *APOE*ε4, eGFR-creatinine, and a comorbidity index, calculated as the total percentage of eight comorbid conditions: obesity, hypertension, diabetes, cancer, ischemic heart disease, chronic heart failure, chronic kidney disease, and chronic obstructive pulmonary disease[66]. Statistical significance was defined at two-sided $p < 0.05$. All statistical analyses were performed in R, version 3.4.1 (R Foundation).

References:

1. Anderson GL, Limacher M, Assaf AR, Bassford T, Beresford SA, Black H *et al.* Effects of conjugated equine estrogen in postmenopausal women with hysterectomy: the Women's Health Initiative randomized controlled trial. *Jama* 2004; **291**(14)**:** 1701-1712.

2. Espeland MA, Rapp SR, Shumaker SA, Brunner R, Manson JE, Sherwin BB *et al.* Conjugated equine estrogens and global cognitive function in postmenopausal women: Women's Health Initiative Memory Study. *Jama* 2004; **291**(24)**:** 2959-2968.

3. Rapp SR, Espeland MA, Shumaker SA, Henderson VW, Brunner RL, Manson JE *et al.* Effect of estrogen plus progestin on global cognitive function in postmenopausal women: the Women's Health Initiative Memory Study: a randomized controlled trial. *Jama* 2003; **289**(20)**:** 2663-2672.

4. Shumaker SA, Legault C, Rapp SR, Thal L, Wallace RB, Ockene JK *et al.* Estrogen plus progestin and the incidence of dementia and mild cognitive impairment in postmenopausal women: the Women's Health Initiative Memory Study: a randomized controlled trial. *Jama* 2003; **289**(20)**:** 2651-2662.

5. Rossouw JE, Anderson GL, Prentice RL, LaCroix AZ, Kooperberg C, Stefanick ML *et al.* Risks and benefits of estrogen plus progestin in healthy postmenopausal women: principal results From the Women's Health Initiative randomized controlled trial. *Jama* 2002; **288**(3)**:** 321-333.

6. Design of the Women's Health Initiative clinical trial and observational study. The Women's Health Initiative Study Group. *Control Clin Trials* 1998; **19**(1)**:** 61-109.

7. Shumaker SA, Reboussin BA, Espeland MA, Rapp SR, McBee WL, Dailey M *et al.* The Women's Health Initiative Memory Study (WHIMS): a trial of the effect of estrogen therapy in preventing and slowing the progression of dementia. *Control Clin Trials* 1998; **19**(6)**:** 604-621.

8. Shumaker SA, Legault C, Kuller L, Rapp SR, Thal L, Lane DS *et al.* Conjugated equine estrogens and incidence of probable dementia and mild cognitive impairment in postmenopausal women: Women's Health Initiative Memory Study. *Jama* 2004; **291**(24)**:** 2947-2958.

9. Patterson RE, Kristal AR, Tinker LF, Carter RA, Bolton MP, Agurs-Collins T. Measurement characteristics of the Women's Health Initiative food frequency questionnaire. *Ann Epidemiol* 1999; **9**(3)**:** 178-187.

10. Anderson GL, Manson J, Wallace R, Lund B, Hall D, Davis S *et al.* Implementation of the Women's Health Initiative study design. *Ann Epidemiol* 2003; **13**(9 Suppl)**:** S5-17.

11. Block G, Woods M, Potosky A, Clifford C. Validation of a self-administered diet history questionnaire using multiple diet records. *J Clin Epidemiol* 1990; **43**(12)**:** 1327-1335.

12. Block G, Hartman AM, Naughton D. A reduced dietary questionnaire: development and validation. *Epidemiology* 1990; **1**(1)**:** 58-64.

13. Shivappa N, Steck SE, Hurley TG, Hussey JR, Hébert JR. Designing and developing a literature-derived, population-based dietary inflammatory index. *Public Health Nutr* 2014; **17**(8)**:** 1689-1696.

14. Shivappa N, Steck SE, Hurley TG, Hussey JR, Ma Y, Ockene IS *et al.* A population-based dietary inflammatory index predicts levels of C-reactive protein in the Seasonal Variation of Blood Cholesterol Study (SEASONS). *Public Health Nutr* 2014; **17**(8)**:** 1825-1833.

15. Tabung FK, Steck SE, Zhang J, Ma Y, Liese AD, Agalliu I *et al.* Construct validation of the dietary inflammatory index among postmenopausal women. *Ann Epidemiol* 2015; **25**(6)**:** 398-405.

16. Shivappa N, Hebert JR, Marcos A, Diaz LE, Gomez S, Nova E *et al.* Association between dietary inflammatory index and inflammatory markers in the HELENA study. *Mol Nutr Food Res* 2017; **61**(6).

17. Shivappa N, Hébert JR, Rietzschel ER, De Buyzere ML, Langlois M, Debruyne E *et al.* Associations between dietary inflammatory index and inflammatory markers in the Asklepios Study. *Br J Nutr* 2015; **113**(4)**:** 665-671.

18. Corley J, Shivappa N, Hébert JR, Starr JM, Deary IJ. Associations between Dietary Inflammatory Index Scores and Inflammatory Biomarkers among Older Adults in the Lothian Birth Cohort 1936 Study. *J Nutr Health Aging* 2019; **23**(7)**:** 628-636.

19. Hayden KM, Beavers DP, Steck SE, Hebert JR, Tabung FK, Shivappa N *et al.* The association between an inflammatory diet and global cognitive function and incident dementia in older women: The Women's Health Initiative Memory Study. *Alzheimers Dement* 2017; **13**(11)**:** 1187-1196.

20. Zabetian-Targhi F, Srikanth VK, Smith KJ, Oddy Ph DW, Beare R, Moran C *et al.* Associations Between the Dietary Inflammatory Index, Brain Volume, Small Vessel Disease, and Global Cognitive Function. *J Acad Nutr Diet* 2021; **121**(5)**:** 915-924 e913.

21. Ferreira M, Cronjé HT, van Zyl T, Bondonno NP, Pieters M. The association between an energy-adjusted dietary inflammatory index and inflammation in rural and urban Black South Africans. *Public Health Nutr* 2021**:** 1-29.

22. Kotemori A, Sawada N, Iwasaki M, Yamaji T, Shivappa N, Hebert JR *et al.* Dietary Inflammatory Index Is Associated With Inflammation in Japanese Men. *Frontiers in Nutrition* 2021; **8**(139).

23. Rafiee P, Shivappa N, Hébert JR, Jaafari Nasab S, Bahrami A, Hekmatdoost A *et al.* Dietary Inflammatory Index and Odds of Colorectal Cancer and Colorectal Adenomatous Polyps in a Case-Control Study from Iran. *Nutrients* 2019; **11**(6)**:** 1213.

24. Teng EL, Chui HC. The Modified Mini-Mental State (3MS) examination. *J Clin Psychiatry* 1987; **48**(8)**:** 314-318.

25. Morris JC, Heyman A, Mohs RC, Hughes JP, van Belle G, Fillenbaum G *et al.* The Consortium to Establish a Registry for Alzheimer's Disease (CERAD). Part I. Clinical and neuropsychological assessment of Alzheimer's disease. *Neurology* 1989; **39**(9)**:** 1159-1165.

26. Folstein MF, Folstein SE, McHugh PR. "Mini-mental state". A practical method for grading the cognitive state of patients for the clinician. *J Psychiatr Res* 1975; **12**(3)**:** 189-198.

27. Reitan RM. Validity of the Trail Making Test as an Indicator of Organic Brain Damage. *Perceptual and Motor Skills* 1958; **8**(3)**:** 271-276.

28. Spitzer RL, Williams JB, Kroenke K, Linzer M, deGruy FV, 3rd, Hahn SR *et al.* Utility of a new procedure for diagnosing mental disorders in primary care. The PRIME-MD 1000 study. *JAMA* 1994; **272**(22)**:** 1749-1756.

29. Yesavage JA, Sheikh JI. Geriatric Depression Scale (GDS). *Clinical Gerontologist* 1986; **5**(1-2)**:** 165-173.

30. Burke WJ, Roccaforte WH, Wengel SP. The short form of the Geriatric Depression Scale: a comparison with the 30-item form. *J Geriatr Psychiatry Neurol* 1991; **4**(3)**:** 173-178.

31. Petersen RC, Stevens JC, Ganguli M, Tangalos EG, Cummings JL, DeKosky ST. Practice parameter: early detection of dementia: mild cognitive impairment (an evidence-based review). Report of the Quality Standards Subcommittee of the American Academy of Neurology. *Neurology* 2001; **56**(9)**:** 1133-1142.

32. *Diagnostic and statistical manual of mental disorders, 4th ed*. American Psychiatric Publishing, Inc.: Arlington, VA, US, 1994, xxvii, 886-xxvii, 886pp.

33. Brandt J, Spencer M, Folstein M. The Telephone Interview for Cognitive Status. *Cognitive and Behavioral Neurology* 1988; **1**(2)**:** 111-118.

34. Ricker JH, Axelrod BN. Analysis of an Oral Paradigm for the Trail Making Test. *Assessment* 1994; **1**(1)**:** 47-52.

35. Gfeller JD, Horn GJ. The East Boston Memory Test: a clinical screening measure for memory impairment in the elderly. *J Clin Psychol* 1996; **52**(2)**:** 191-196.

36. Wechsler D. *Wechsler Memory Scale, Revised*. Psychological Corporation: San Antonio, TX, 1987.

37. Benton AL. Differential behavioral effects in frontal lobe disease. *Neuropsychologia* 1968; **6**(1)**:** 53-60.

38. Kawas C, Segal J, Stewart WF, Corrada M, Thal LJ. A validation study of the Dementia Questionnaire. *Arch Neurol* 1994; **51**(9)**:** 901-906.

39. Levine DW, Lewis MA, Bowen DJ, Kripke DF, Kaplan RM, Naughton MJ *et al.* Reliability and validity of Women's Health Initiative Insomnia Rating Scale. *Psychological Assessment* 2003; **15:** 137-148.

40. Walker KA, Chen J, Zhang J, Fornage M, Yang Y, Zhou L *et al.* Large-scale plasma proteomic analysis identifies proteins and pathways associated with dementia risk. *Nature Aging* 2021; **1**(5)**:** 473-489.

41. The Atherosclerosis Risk in Communities (ARIC) Study: design and objectives. The ARIC investigators. *Am J Epidemiol* 1989; **129**(4)**:** 687-702.

42. Tin A, Yu B, Ma J, Masushita K, Daya N, Hoogeveen RC *et al.* Reproducibility and Variability of Protein Analytes Measured Using a Multiplexed Modified Aptamer Assay. *J Appl Lab Med* 2019; **4**(1)**:** 30-39.

43. Knopman DS, Gottesman RF, Sharrett AR, Tapia AL, DavisThomas S, Windham BG *et al.* Midlife vascular risk factors and midlife cognitive status in relation to prevalence of mild cognitive impairment and dementia in later life: The Atherosclerosis Risk in Communities Study. *Alzheimers Dement* 2018; **14**(11)**:** 1406-1415.

44. *Diagnostic and statistical manual of mental disorders: DSM-5™, 5th ed*. American Psychiatric Publishing, Inc.: Arlington, VA, US, 2013, xliv, 947-xliv, 947pp.

45. Carpenter CR, DesPain B, Keeling TN, Shah M, Rothenberger M. The Six-Item Screener and AD8 for the detection of cognitive impairment in geriatric emergency department patients. *Ann Emerg Med* 2011; **57**(6)**:** 653-661.

46. Galvin JE, Roe CM, Xiong C, Morris JC. Validity and reliability of the AD8 informant interview in dementia. *Neurology* 2006; **67**(11)**:** 1942-1948.

47. Shock NW, Greulich RC, Aremberg D, Costa PT, Lakatta EG, Tobin JD. *Normal human aging: The Baltimore longitudinal study of aging*. National Institutes of Health: Washington, D.C., 1984.

48. Varadaraj V, Munoz B, Deal JA, An Y, Albert MS, Resnick SM *et al.* Association of Vision Impairment With Cognitive Decline Across Multiple Domains in Older Adults. *JAMA Netw Open* 2021; **4**(7)**:** e2117416-e2117416.

49. Armstrong NM, An Y, Doshi J, Erus G, Ferrucci L, Davatzikos C *et al.* Association of Midlife Hearing Impairment With Late-Life Temporal Lobe Volume Loss. *JAMA Otolaryngol Head Neck Surg* 2019; **145**(9)**:** 794-802.

50. Armstrong NM, An Y, Shin JJ, Williams OA, Doshi J, Erus G *et al.* Associations between cognitive and brain volume changes in cognitively normal older adults. *Neuroimage* 2020; **223:** 117289.

51. Doshi J, Erus G, Ou Y, Resnick SM, Gur RC, Gur RE *et al.* MUSE: MUlti-atlas region Segmentation utilizing Ensembles of registration algorithms and parameters, and locally optimal atlas selection. *Neuroimage* 2016; **127:** 186-195.

52. Davatzikos C, Genc A, Xu D, Resnick SM. Voxel-based morphometry using the RAVENS maps: methods and validation using simulated longitudinal atrophy. *Neuroimage* 2001; **14**(6)**:** 1361-1369.

53. Driscoll I, Beydoun MA, An Y, Davatzikos C, Ferrucci L, Zonderman AB *et al.* Midlife obesity and trajectories of brain volume changes in older adults. *Hum Brain Mapp* 2012; **33**(9)**:** 2204-2210.

54. Lee S, Zipunnikov V, Reich DS, Pham DL. Statistical image analysis of longitudinal RAVENS images. *Frontiers in Neuroscience* 2015; **9**.

55. Davatzikos C, Xu F, An Y, Fan Y, Resnick SM. Longitudinal progression of Alzheimer's-like patterns of atrophy in normal older adults: the SPARE-AD index. *Brain* 2009; **132**(Pt 8)**:** 2026-2035.

56. Davatzikos C, Bhatt P, Shaw LM, Batmanghelich KN, Trojanowski JQ. Prediction of MCI to AD conversion, via MRI, CSF biomarkers, and pattern classification. *Neurobiol Aging* 2011; **32**(12)**:** 2322.e2319-2327.

57. Fan Y, Shen D, Gur RC, Gur RE, Davatzikos C. COMPARE: classification of morphological patterns using adaptive regional elements. *IEEE transactions on medical imaging* 2006; **26**(1)**:** 93-105.

58. Davatzikos C, Resnick SM, Wu X, Parmpi P, Clark CM. Individual patient diagnosis of AD and FTD via high-dimensional pattern classification of MRI. *Neuroimage* 2008; **41**(4)**:** 1220-1227.

59. Toledo JB, Weiner MW, Wolk DA, Da X, Chen K, Arnold SE *et al.* Neuronal injury biomarkers and prognosis in ADNI subjects with normal cognition. *Acta neuropathologica communications* 2014; **2**(1)**:** 1-9.

60. Da X, Toledo JB, Zee J, Wolk DA, Xie SX, Ou Y *et al.* Integration and relative value of biomarkers for prediction of MCI to AD progression: spatial patterns of brain atrophy, cognitive scores, APOE genotype and CSF biomarkers. *NeuroImage: Clinical* 2014; **4:** 164-173.

61. Cortes C, Vapnik V. Support-vector networks. *Machine Learning* 1995; **20**(3)**:** 273-297.

62. Vapnik V. *The nature of statistical learning theory*. Springer science & business media1999.

63. Trares K, Bhardwaj M, Perna L, Stocker H, Petrera A, Hauck SM *et al.* Association of the inflammation-related proteome with dementia development at older age: results from a large, prospective, population-based cohort study. *Alzheimers Res Ther* 2022; **14**(1)**:** 128.

64. Lee CH, Cook S, Lee JS, Han B. Comparison of Two Meta-Analysis Methods: Inverse-Variance-Weighted Average and Weighted Sum of Z-Scores. *Genomics Inform* 2016; **14**(4)**:** 173-180.

65. Kivimäki M, Walker KA, Pentti J, Nyberg ST, Mars N, Vahtera J *et al.* Cognitive stimulation in the workplace, plasma proteins, and risk of dementia: three analyses of population cohort studies. *Bmj* 2021; **374:** n1804.

66. Seliger SL, Wendell CR, Waldstein SR, Ferrucci L, Zonderman AB. Renal function and long-term decline in cognitive function: the Baltimore Longitudinal Study of Aging. *Am J Nephrol* 2015; **41**(4-5)**:** 305-312.

SUPPLEMENTARY TABLES

**Title:** Plasma proteins related to inflammatory diet predict future cognitive impairment	
**Authors**: Michael R. Duggan PhD, Lauren Butler BS, Zhongsheng Peng MD, PhD, Gulzar, N. Daya MS, MS, Abhay Moghekar MBBS, Yang An MS, Stephen R. Rapp PhD, Kathleen M. Hayden PhD, Aladdin H. Shadyab PhD, Ginny Natale PhD, Longjian Liu MD, PhD, Linda Snetselaar PhD, Ruin Moaddel PhD, Casey M. Rebholz PhD, Kevin Sullivan PhD, Christie M. Ballantyne MD, Susan M. Resnick PhD, Luigi Ferrucci MD, PhD, Keenan A. Walker PhD	
**Table**	**Title**
Supplementary Table 1	List of proteins in each Olink panel
Supplementary Table 2	Proteins excluded form analyses due to >25% invalid samples
Supplementary Table 3	Frequencies of imputed values, intra-assay and inter-assay CVs per protein
Supplementary Table 4	Full list of proteins annotated in each immune/inflammatory pathway
Supplementary Table 5	Association between levels of inflammatory/immune proteins with EDII scores (partial Spearman correlation's (adjusted for age))
Supplementary Table 6	Association between immune/inflammatory pathway composite scores with EDII scores (partial Spearman correlation's (adjusted for age))
Supplementary Table 7	Multivariable logistic regression models of EDII-related proteins with odds of cognitive impairment
Supplementary Table 8	Biological process, molecular functions, and diseases associated with each inflammatory diet candidate protein.
Supplementary Table 9	Multivariable logistic regression models of EDII-related immune/inflammatory pathways with odds of cognitive impairment
Supplementary Table 10	Fixed effect meta-analysis examining candidate proteins using cohort specific effect estimates WHIMS and ESTHER
Supplementary Table 11	ARIC participant characteristics stratified according to late-life baseline (2011-2013) cognitive status
Supplementary Table 12	Correlations of candidate protein measurements across Olink® and Somscan® platforms (Spearman's)
Supplementary Table 13	Association of inflammatory diet proteins with time-to-dementia onset in ARIC
Supplementary Table 14	Correlations of variables related to EDII as well as cognitive impairment with plasma biomarkers of AD pathology (AB42/40) and neurodegeneration (NfL) (partial Spearman correlation's (adjusted for age))
Supplementary Table 15	BLSA participant characteristics stratified according to low and high SPARE-AD groups (median split)
Supplementary Table 16	Associations of inflammatory diet proteins with the SPARE-AD in the BLSA
Supplementary Table 17	Consensus transcript expression levels (normalized Transcripts per Million; nTPM) of genes encoding for candidate proteins across immune tissues and cells of interest
Supplementary Table 18	Differential expression of genes encoding for candidate proteins (measured via RNA-seq) following in vitro immune challenge (LPS Treatment)

Supplementary Table 19	Gene Enrichment analyses using PANTHER functional classification platform
Supplementary Table 20	Protein-Protein interaction network analyses powered by the STRING database
Supplementary Table 21	Therapeutic drugs targeting candidate proteins of interest

Supplementary Table 1. List of proteins in each Olink panel			
**Olink Target 96 Inflammation**		**Olink Target 96 Immune Response**	
Protein	Full Name	Protein	Full Name
ADA	Adenosine Deaminase (ADA)	MILR1	Allergin-1 (MILR1)
ARTN	Artemin (ARTN)	AREG	Amphiregulin (AREG)
AXIN1	Axin-1 (AXIN1)	ARNT	Aryl hydrocarbon receptor nuclear translocator (ARNT)
Beta_NGF	Beta-nerve growth factor (Beta-NGF)	BIRC2	Baculoviral IAP repeat-containing protein 2 (BIRC2)
CCL19	C-C motif chemokine 19 (CCL19)	GLB1	Beta-galactosidase (GLB1)
CCL20	C-C motif chemokine 20 (CCL20)	BTN3A2	Butyrophilin subfamily 3 member A2 (BTN3A2)
CCL23	C-C motif chemokine 23 (CCL23)	CLEC4A	C-type lectin domain family 4 member A (CLEC4A)
CCL25	C-C motif chemokine 25 (CCL25)	CLEC4C	C-type lectin domain family 4 member C (CLEC4C)
CCL28	C-C motif chemokine 28 (CCL28)	CLEC4D	C-type lectin domain family 4 member D (CLEC4D)
CCL3	C-C motif chemokine 3 (CCL3)	CLEC4G	C-type lectin domain family 4 member G (CLEC4G)
CCL4	C-C motif chemokine 4 (CCL4)	CLEC6A	C-type lectin domain family 6 member A (CLEC6A)
CXCL1	C-X-C motif chemokine 1 (CXCL1)	CLEC7A	C-type lectin domain family 7 member A (CLEC7A)
CXCL10	C-X-C motif chemokine 10 (CXCL10)	CD83	CD83 antigen (CD83)
CXCL11	C-X-C motif chemokine 11 (CXCL11)	CNTNAP2	Contactin-associated protein-like 2 (CNTNAP2)
CXCL5	C-X-C motif chemokine 5 (CXCL5)	CDSN	Corneodesmosin (CDSN)
CXCL6	C-X-C motif chemokine 6 (CXCL6)	HSD11B1	Corticosteroid 11-beta-dehydrogenase isozyme 1 (HSD11B1)
CXCL9	C-X-C motif chemokine 9 (CXCL9)	CXADR	Coxsackievirus and adenovirus receptor (CXADR)
CASP_8	Caspase-8 (CASP-8)	CKAP4	Cytoskeleton-associated protein 4 (CKAP4)
CD40	CD40L receptor (CD40)	DGKZ	Diacylglycerol kinase zeta (DGKZ)
CDCP1	CUB domain-containing protein 1 (CDCP1)	DCBLD2	Discoidin, CUB and LCCL domain-containing protein 2 (DCBLD2)
CST5	Cystatin D (CST5)	DFFA	DNA fragmentation factor subunit alpha (DFFA)

DNER	Delta and Notch-like epidermal growth factor-related receptor (DNER)	DAPP1	Dual adapter for phosphotyrosine and 3-phosphotyrosine and 3-phosphoinositide (DAPP1)
CCL11	Eotaxin (CCL11)	DCTN1	Dynactin subunit 1 (DCTN1)
v4E_BP1	Eukaryotic translation initiation factor 4E-binding protein 1 (4E-BP1)	TRIM21	E3 ubiquitin-protein ligase TRIM21 (TRIM21)
FGF_19	Fibroblast growth factor 19 (FGF-19)	EGLN1	Egl nine homolog 1 (EGLN1)
FGF_21	Fibroblast growth factor 21 (FGF21)	CCL11	Eotaxin (CCL11)
FGF_23	Fibroblast growth factor 23 (FGF-23)	EIF4G1	Eukaryotic translation initiation factor 4 gamma 1 (EIF4G1)
FGF_5	Fibroblast growth factor 5 (FGF-5)	EIF5A	Eukaryotic translation initiation factor 5A-1 (EIF5A)
Flt3L	Fms-related tyrosine kinase 3 ligand (Flt3L)	FCRL3	Fc receptor-like protein 3 (FCRL3)
CX3CL1	Fractalkine (CX3CL1)	FCRL6	Fc receptor-like protein 6 (FCRL6)
GDNF	Glial cell line-derived neurotrophic factor (GDNF)	FGF2	Fibroblast growth factor 2 (FGF2)
HGF	Hepatocyte growth factor (HGF)	FXYD5	FXYD domain-containing ion transport regulator 5 (FXYD5)
IFN_gamma	Interferon gamma (IFN-gamma)	HCLS1	Hematopoietic lineage cell-specific protein (HCLS1)
IL_1_alpha	Interleukin-1 alpha (IL-1 alpha)	HNMT	Histamine N-methyltransferase (HNMT)
IL10	Interleukin-10 (IL10)	KPNA1	Importin subunit alpha-5 (KPNA1)
IL_10RA	Interleukin-10 receptor subunit alpha (IL-10RA)	DPP10	Inactive dipeptidyl peptidase 10 (DPP10)
IL_10RB	Interleukin-10 receptor subunit beta (IL-10RB)	ITM2A	Integral membrane protein 2A (ITM2A)
IL_12B	Interleukin-12 subunit beta (IL-12B)	ITGA11	Integrin alpha-11 (ITGA11)
IL13	Interleukin-13 (IL-13)	ITGA6	Integrin alpha-6 (ITGA6)
IL_15RA	Interleukin-15 receptor subunit alpha (IL-15RA)	ITGB6	Integrin beta-6 (ITGB6)
IL_17A	Interleukin-17A (IL-17A)	IFNLR1	Interferon lambda receptor 1 (IFNLR1)
IL_17C	Interleukin-17C (IL-17C)	IRF9	Interferon regulatory factor 9 (IRF9)
IL18	Interleukin-18 (IL-18)	IRAK1	Interleukin-1 receptor-associated kinase 1 (IRAK1)
IL_18R1	Interleukin-18 receptor 1 (IL-18R1)	IRAK4	Interleukin-1 receptor-associated kinase 4 (IRAK4)

IL2	Interleukin-2 (IL-2)	IL10	Interleukin-10 (IL10)
IL_2RB	Interleukin-2 receptor subunit beta (IL-2RB)	IL12RB1	Interleukin-12 receptor subunit beta-1 (IL12RB1)
IL_20	Interleukin-20 (IL-20)	IL5	Interleukin-5 (IL5)
IL_20RA	Interleukin-20 receptor subunit alpha (IL-20RA)	IL6	Interleukin-6 (IL6)
IL_22_RA1	Interleukin-22 receptor subunit alpha-1 (IL-22 RA1)	ICA1	Islet cell autoantigen 1 (ICA1)
IL_24	Interleukin-24 (IL-24)	KRT19	Keratin, type I cytoskeletal 19 (KRT19)
IL33	Interleukin-33 (IL-33)	LILRB4	Leukocyte immunoglobulin-like receptor subfamily B member 4 (LILRB4)
IL4	Interleukin-4 (IL-4)	LAG3	Lymphocyte activation gene 3 protein (LAG3)
IL5	Interleukin-5 (IL5)	LY75	Lymphocyte antigen 75 (LY75)
IL6	Interleukin-6 (IL6)	LAMP3	Lysosome-associated membrane glycoprotein 3 (LAMP3)
IL7	Interleukin-7 (IL-7)	MASP1	Mannan-binding lectin serine protease 1 (MASP1)
IL8	Interleukin-8 (IL-8)	NF2	Merlin (NF2)
LAP_TGF_beta_1	Latency-associated peptide transforming growth factor beta-1 (LAP TGF-beta-1)	MGMT	Methylated-DNA--protein-cysteine methyltransferase (MGMT)
LIF	Leukemia inhibitory factor (LIF)	NCR1	Natural cytotoxicity triggering receptor 1 (NCR1)
LIF_R	Leukemia inhibitory factor receptor (LIF-R)	KLRD1	Natural killer cells antigen CD94 (KLRD1)
CSF_1	Macrophage colony-stimulating factor 1 (CSF-1)	PPP1R9B	Neurabin-2 (PPP1R9B)
MMP_1	Matrix metalloproteinase-1 (MMP-1)	NTF4	Neurotrophin-4 (NTF4)
MMP_10	Matrix metalloproteinase-10 (MMP-10)	NFATC3	Nuclear factor of activated T-cells, cytoplasmic 3 (NFATC3)
MCP_1	Monocyte chemotactic protein 1 (MCP-1)	PTH1R	Parathyroid hormone/parathyroid hormone-related peptide receptor (PTH1R)
MCP_2	Monocyte chemotactic protein 2 (MCP-2)	PSIP1	PC4 and SFRS1-interacting protein (PSIP1)
MCP_3	Monocyte chemotactic protein 3 (MCP-3)	PRDX1	Peroxiredoxin-1 (PRDX1)
MCP_4	Monocyte chemotactic protein 4 (MCP-4)	PRDX5	Peroxiredoxin-5, mitochondrial (PRDX5)

CD244	Natural killer cell receptor 2B4 (CD244)	PIK3AP1	Phosphoinositide 3-kinase adapter protein 1 (PIK3AP1)
NT_3	Neurotrophin-3 (NT-3)	PLXNA4	Plexin-A4 (PLXNA4)
NRTN	Neurturin (NRTN)	GALNT3	Polypeptide N-acetylgalactosaminyltransferase 3 (GALNT3)
OSM	Oncostatin-M (OSM)	DDX58	Probable ATP-dependent RNA helicase DDX58 (DDX58)
OPG	Osteoprotegerin (OPG)	FAM3B	Protein FAM3B (FAM3B)
PD_L1	Programmed cell death 1 ligand 1 (PD-L1)	HEXIM1	Protein HEXIM1 (HEXIM1)
EN_RAGE	Protein S100-A12 (EN-RAGE)	PRKCQ	Protein kinase C theta type (PRKCQ)
SLAMF1	Signaling lymphocytic activation molecule (SLAMF1)	SPRY2	Protein sprouty homolog 2 (SPRY2)
SIRT2	SIR2-like protein 2 (SIRT2)	PADI2	Protein-arginine deiminase type-2 (PADI2)
STAMBP	STAM-binding protein (STAMBP)	SH2D1A	SH2 domain-containing protein 1A (SH2D1A)
SCF	Stem cell factor (SCF)	SH2B3	SH2B adapter protein 3 (SH2B3)
ST1A1	Sulfotransferase 1A1 (ST1A1)	SIT1	Signaling threshold-regulating transmembrane adapter 1 (SIT1)
CD6	T cell surface glycoprotein CD6 isoform (CD6)	SRPK2	SRSF protein kinase 2 (SRPK2)
CD5	T-cell surface glycoprotein CD5 (CD5)	STC1	Stanniocalcin-1 (STC1)
CD8A	T-cell surface glycoprotein CD8 alpha chain (CD8A)	CXCL12	Stromal cell-derived factor 1 (CXCL12)
TSLP	Thymic stromal lymphopoietin (TSLP)	CD28	T-cell-specific surface glycoprotein CD28 (CD28)
TNFB	TNF-beta (TNFB)	PRDX3	Thioredoxin-dependent peroxide reductase, mitochondrial (PRDX3)
TRANCE	TNF-related activation-induced cytokine (TRANCE)	TRAF2	TNF receptor-associated factor 2 (TRAF2)
TRAIL	TNF-related apoptosis-inducing ligand (TRAIL)	TANK	TRAF family member-associated NF-kappa-B activator (TANK)
TGF_alpha0	Transforming growth factor alpha (TGF-alpha)	JUN	Transcription factor AP-1 (JUN)
TWEAK	Tumor necrosis factor (Ligand) superfamily, member 12 (TWEAK)	BACH1	Transcription regulator protein BACH1 (BACH1)
TNF	Tumor necrosis factor (TNF)	TREM1	Triggering receptor expressed on myeloid cells 1 (TREM1)

TNFSF14	Tumor necrosis factor ligand superfamily member 14 (TNFSF14)	TRIM5	Tripartite motif-containing protein 5 (TRIM5)
TNFRSF9	Tumor necrosis factor receptor superfamily member 9 (TNFRSF9)	TPSAB1	Tryptase alpha/beta-1 (TPSAB1)
uPA	Urokinase-type plasminogen activator (uPA)	EDAR	Tumor necrosis factor receptor superfamily member EDAR (EDAR)
VEGFA	Vascular endothelial growth factor A (VEGF-A)	ZBTB16	Zinc finger and BTB domain-containing protein 16 (ZBTB16)

**Supplementary Table 2.** Proteins excluded form analyses due to >25% invalid samples	
**Protein**	**Number of samples below LOD or excluded due to quality control warning**
ARNT	1387
ARTN	1286
Beta_NGF	1117
BIRC2	711
DGKZ	778
EIF5A	1441
FXYD5	624
GALNT3	463
GLB1	959
IL_1_alpha	1484
IL_17C	410
IL_20	1154
IL_20RA	530
IL_22_RA1	874
IL_24	1340
IL_2RB	1168
IL13	1321
IL2	1522
IL33	1416
IL4	1044
IL5	1066
JUN0	1341
KPNA1	1225
LIF	1282
NRTN	1056
PAD12	396
PRKCQ	542
SLAMF1	588
TSLP	1440

Supplementary Table 3. Frequencies of imputed values per protein

Protein	Number of imputed values	Intra Assay CV	Inter Assay CV
ADA	2	2.096	4.596
AREG	5	3.132	2.902
AXIN1	8	3.471	-0.048
BACH1	60	4.911	0.836
BTN3A2	5	3.189	2.418
CASP_8	25	4.690	0.822
CCL11	5	1.395	6.241
CCL19	2	1.245	7.647
CCL20	2	2.039	5.958
CCL23	2	1.200	8.645
CCL25	2	1.653	5.250
CCL28	2	5.416	1.435
CCL3	2	2.059	3.442
CCL4	2	1.665	4.500
CD244	2	1.763	6.370
CD28	297	5.521	1.019
CD40	3	1.027	10.192
CD5	2	1.750	4.369
CD6	2	1.986	5.156
CD83	5	3.330	3.071
CD8A	2	1.076	9.331
CDCP1	2	2.690	2.338
CDSN	143	2.714	2.827
CKAP4	5	2.843	3.909
CLEC4A	6	2.400	4.123
CLEC4C	6	2.337	3.533
CLEC4D	5	4.181	1.844
CLEC4G	6	2.712	2.985
CLEC6A	8	6.036	2.168
CLEC7A	14	3.041	2.965
CNTNAP2	8	4.163	1.363
CSF_1	2	0.823	9.112
CST5	2	1.196	4.762
CX3CL1	2	1.940	5.076
CXADR	5	4.873	1.795
CXCL1	2	1.135	4.721
CXCL10	2	1.229	8.610
CXCL11	2	1.833	6.323
CXCL12	105	6.666	0.941

CXCL5	2	1.076	5.454
CXCL6	2	1.450	6.353
CXCL9	2	1.286	6.426
DAPP1	26	1.809	1.968
DCBLD2	5	0.963	8.072
DCTN1	6	2.782	1.449
DDX58	20	3.905	2.434
DFFA	5	2.390	3.331
DNER	2	0.926	8.384
DPP10	7	5.581	1.347
EDAR	58	3.799	1.339
EGLN1	198	5.791	1.075
EIF4G1	5	2.516	1.367
EN_RAGE	2	6.018	2.706
FAM3B	5	2.104	4.346
FCRL3	73	4.570	1.680
FCRL6	5	3.016	3.351
FGF_19	2	1.442	7.112
FGF_21	3	1.759	4.228
FGF_23	2	3.714	1.912
FGF_5	47	11.296	0.095
FGF2	129	5.115	0.479
Flt3L	2	1.013	8.547
GDNF	7	6.019	1.818
HCLS1	5	2.787	2.764
HEXIM1	5	2.620	3.029
HGF	2	1.118	7.865
HNMT	5	1.176	8.677
HSD11B1	5	3.252	2.984
ICA1	209	5.007	0.623
IFN_gamma	4	2.063	5.606
IFNLR1	5	4.450	2.162
IL_10RA	101	9.827	0.390
IL_10RB	2	1.641	5.182
IL_12B	2	1.487	4.973
IL_15RA	3	8.060	0.693
IL_17A	57	6.177	1.439
IL_18R1	2	1.075	7.389
IL10	7	3.240	3.043
IL12RB1	5	4.382	2.005
IL18	2	1.216	7.591

IL6	6	2.595	1.969
IL7	2	3.004	1.585
IL8	2	2.059	4.246
IRAK1	24	3.669	0.486
IRAK4	70	2.556	0.742
IRF9	279	3.155	1.311
ITGA11	5	3.054	2.773
ITGA6	44	7.889	0.986
ITGB6	5	4.231	3.181
ITM2A	5	4.089	3.881
KLRD1	5	1.537	6.324
KRT19	56	3.391	3.321
LAG3	5	2.973	2.735
LAMP3	5	2.066	4.044
LAP_TGF_beta_1	2	1.922	6.222
LIF_R	2	2.687	3.135
LILRB4	5	2.503	3.017
LY75	7	3.639	2.240
MASP1	5	2.927	2.291
MCP_1	2	0.956	11.889
MCP_2	2	1.267	7.557
MCP_3	75	5.021	1.406
MCP_4	2	1.020	12.182
MGMT	5	1.465	2.047
MILR1	5	2.892	2.991
MMP_1	2	1.235	6.522
MMP_10	2	1.037	8.212
NCR1	6	5.741	2.106
NF2	268	16.633	-1.526
NFATC3	92	8.123	0.867
NT_3	2	8.590	1.819
NTF4	12	8.866	1.577
OPG	2	0.855	9.474
OSM	2	4.000	3.204
PD_L1	2	1.834	5.711
PIK3AP1	5	3.102	1.372
PLXNA4	5	2.896	2.603
PPP1R9B	11	3.249	1.053
PRDX1	45	5.271	0.642
PRDX3	215	13.657	-0.516
PRDX5	5	2.778	2.222

PSIP1	22	5.177	1.331
PTH1R	24	6.581	3.904
SCF0	2	0.805	8.572
SH2B3	21	3.279	6.476
SH2D1A	21	4.060	2.237
SIRT2	4	3.565	0.755
SIT1	6	6.099	3.724
SPRY2	125	4.512	1.493
SRPK2	201	5.206	0.527
ST1A1	24	3.207	0.600
STAMBP	2	3.340	2.578
STC1	5	1.669	6.574
TANK	140	4.342	1.583
TGF_alpha	2	3.090	2.718
TNF	2	2.689	2.240
TNFB	2	2.242	4.066
TNFRSF9	2	1.396	6.277
TNFSF14	2	3.268	2.583
TPSAB1	6	2.203	4.459
TRAF2	9	2.928	1.425
TRAIL	2	0.972	7.560
TRANCE	2	2.070	4.475
TREM1	5	35.380	-0.236
TRIM21	31	4.020	1.442
TRIM5	70	3.501	0.832
TWEAK	2	1.157	8.726
uPA	2	0.998	9.376
v4E_BP1	2	1.914	4.481
VEGFA	2	1.111	9.399
ZBTB160	35	3.670	0.550
Average	25.66	3.525	3.765
SD	55.35	3.544	2.768
Median	5	2.843	2.985
Minimum	2	0.805	0.048
Maximum	297	35.380	3.765

Supplementary Table 4. Full list of proteins annotated in each immune/inflammatory pathway	
Apoptotic process (GO:0006915)	ADA AREG AXIN1 CASP8 CCL19 CD28 CD40 CD5 CX3CL1 CXCL10 CXCL12 DFFA EDAR FAM3B GDNF HCLS1 HGF IFN_gamma IL_12B IL_17A IL10 IL6 IL7 ITGA6 LAMP3 LAP_TGF_beta_1 MGMT NF2 NT_3 NFT4 OPG PD_L1 PRDX3 PRDX5 SCF0 SIRT2 SPRY2 SRPK2 STAMBP TGF_alpha TNF TNFB TNFRSF9 TRAF2 TRAIL VEGFA ZBTB16 CCL3 FGF_21 MCP_1
Cell activation in immune response (GO:0002253)	ADA CD28 SH2D1A TRIM5
Cell adhesion (GO:0007155)	ADA CCL11 CCL19 CCL25 CCL28 CCL4 CD28 CDSN CLEC4A CLEC4G CLEC7A CNTNAP2 CSF_1 CX3CL1 CXADR CXCL12 IFN_gamma IL_12B IL10 IL12RB1 IL6 IL7 IL8 IRAK1 ITGA11 MILR1 NF2 SCF0 TNF TRANCE VEGFA ZBTB16 IL18 MCP_1 uPAO
Response to cytokine stimulus (GO:0071345)	CSF_1 CXCL12 IFN_gamma IL_12B IL12RB1 IL6 IL7 IL8 IRAK1 TNF TRANCE CCL3 FGF_23 IL18 MCP_1
Chemotaxis (GO:0006935)	CSF_1 CXCL12 IL6 IL8 TRANCE CCL3 MCP_1 uPAO
Extracellular matrix organization (GO:0030198)	IL6 TNF
Inflammatory response (GO:0006954)	ADA CD28 CSF_1 IFN_gamma IL_10RB IL_12B IL_17A IL_18R1 IL10 IL6 IL8 NFATC3 TNF TNFB TRANCE TREM1 IL18 MCP_1
MAPK cascade (GO:0000165)	FGF_19 IL6 IRAK1 IRAK4 NT_3 PRDX1 SCF0 TGF_alpha TNF TRANCE TRIM5 VEGFA FGF_21 FGF_23 MCP_1
Regulation of immune response (GO:0050776)	ADA CD28 IFN_gamma IL_12B IL_17A IL_18R1 IL10 IL12RB1 IL6 IRAK1 IRAK4 NCR1 SH2D1A TNF TNFB TRIM21 TRIM5 IL18
Response to hypoxia (GO:0001666)	ADA CXCL12 IRAK1 SIRT2 TNF TNFB VEGFA uPAO
Secretion (GO:0046903)	ADA CCL28 CCL4 CXCL12 FAM3B FGF_19 GDNF HEXIM1 ICA1 IFN_gamma IL_12B IL6 LAP_TGF_beta_1 LIF_R MCP_2 MILR1 OSM STC1 TNF TRANCE VEGFA FGF_23

Supplementary Table 5. Association between levels of inflammatory/immune proteins with EDII scores (partial Spearman correlation's (adjusted for age))

Protein	rho	p	FDR
LILRB4	0.148	<.001	0.0000
FGF-21	0.143	<.001	0.0000
STC1	0.131	<.001	0.0000
IL-6	0.128	<.001	0.0000
CD8A	0.127	<.001	0.0000
IFNLR1	0.127	<.001	0.0000
AREG	0.126	<.001	0.0000
TREM1	0.125	<.001	0.0000
CD83	0.119	<.001	0.0001
IL-18R1	0.117	<.001	0.0001
CKAP4	0.113	<.001	0.0002
HNMT	0.113	<.001	0.0002
FGF-23	0.112	<.001	0.0002
CLEC6A	0.108	<.001	0.0002
CSF-1	0.108	<.001	0.0003
TNFRSF9	0.101	<.001	0.0003
HGF	0.099	<.001	0.0009
OPG0	0.099	<.001	0.0011
MASP1	0.098	<.001	0.0011
CLEC4D	0.096	<.001	0.0013
IL-10	0.094	<.001	0.0019
IL-12RB1	0.091	<.001	0.0022
TGF-alpha	0.088	<.001	0.0035
DNER	-0.111	<.001	0.0047
CCL20	0.084	0.001	0.0058
IL-12B	0.083	0.001	0.0058
CCL19	0.079	0.002	0.0104
CD5	0.08	0.002	0.0104
NFATC3	0.082	0.002	0.0104
KLRD1	0.079	0.003	0.0146
VEGFA	0.077	0.003	0.0146
CLEC4G	0.075	0.004	0.0189
FCRL6	0.073	0.005	0.0216
PD-L1	0.074	0.005	0.0216
TNF	0.073	0.005	0.0216
FAM3B	0.072	0.006	0.0238
IL-10RB	0.072	0.006	0.0238
IL-17A	0.072	0.006	0.0238

TWEAK	-0.07	0.007	0.0271
CCL23	0.066	0.011	0.0415
CLEC7A	0.066	0.011	0.0442
IL-15RA	0.062	0.016	0.0575
TPSAB1	0.062	0.017	0.0597
IL-18	0.062	0.018	0.0618
CXCL10	0.059	0.023	0.0772
CCL11	0.059	0.025	0.0788
TRANCE	0.057	0.027	0.0867
ITGA11	-0.056	0.031	0.0975
MCP-3	0.056	0.032	0.0986
CD244	0.054	0.036	0.1087
SIT1	0.055	0.037	0.1095
MCP-10	0.054	0.039	0.1162
CCL3	0.053	0.041	0.1168
MMP-10	0.053	0.044	0.1230
CDCP1	0.051	0.049	0.1345
BTN3A2	0.051	0.051	0.1375
MILR1	0.05	0.054	0.1431
CD28	0.049	0.062	0.1614
CX3CL1	0.048	0.063	0.1634
CD40	0.048	0.065	0.1634
CLEC4A	-0.048	0.067	0.1634
OSM	0.047	0.074	0.1802
TRAIL	0.046	0.08	0.1917
NCR1	0.042	0.112	0.2666
TRAF2	0.041	0.118	0.2741
IL-10RA	0.04	0.127	0.2928
IL-8	0.039	0.135	0.3043
HSD11B1	0.038	0.15	0.3286
GDNF	0.035	0.177	0.3873
LIF-R	-0.035	0.182	0.3948
FGF_19	-0.033	0.199	0.4168
PTH1R	-0.033	0.203	0.4257
DCBLD2	-0.033	0.211	0.4326
LAMP3	0.033	0.211	0.4326
CXCL6	0.031	0.233	0.4709
CXCL9	0.031	0.236	0.4709
DAPP1	-0.031	0.242	0.4746
NT-3	-0.03	0.25	0.4801
KRT19	0.03	0.253	0.4912

TNFSF14	0.029	0.263	0.4917
DDX58	-0.029	0.264	0.4917
CD6	0.029	0.266	0.4917
PSIP1	-0.029	0.275	0.4967
PRDX3	-0.028	0.28	0.5033
CDSN	0.028	0.285	0.5116
SCF	-0.027	0.296	0.5215
LAG3	0.026	0.313	0.5433
MCP-2	0.025	0.338	0.5819
PIK3AP1	-0.025	0.342	0.5819
FGF-5	-0.023	0.37	0.6173
TRIM5	0.024	0.37	0.6173
IFN-gamma	0.023	0.384	0.6352
MCP-4	-0.022	0.393	0.6365
FCRL3	0.022	0.406	0.6490
DPP10	0.021	0.417	0.6596
TNFB	0.021	0.425	0.6701
CASP-8	-0.02	0.436	0.6756
CCL25	0.02	0.442	0.6764
LAP-TGF-beta-1	0.02	0.448	0.6894
ITGA6	-0.019	0.471	0.7112
HCLS1	-0.019	0.478	0.7146
AXIN1	0.018	0.482	0.7165
EDAR	0.017	0.514	0.7550
4E_BP1	0.016	0.527	0.7636
ITM2A	0.016	0.529	0.7636
CLEC4C	0.014	0.586	0.8211
CXCL5	-0.014	0.59	0.8211
CXCL11	0.013	0.605	0.8211
LY75	0.014	0.605	0.8211
ST1A1	0.013	0.607	0.8211
NF2	-0.013	0.61	0.8211
PPP1R9B	-0.013	0.611	0.8211
SRPK2	-0.011	0.666	0.8900
ITGB6	0.01	0.703	0.9270
IRAK4	-0.01	0.704	0.9270
DCTN1	-0.009	0.742	0.9364
NTF4	-0.008	0.746	0.9364
TANK	-0.008	0.747	0.9364
IRF9	-0.008	0.758	0.9364
uPA	-0.008	0.762	0.9364

SIRT2	0.008	0.764	0.9364
MMP-1	-0.008	0.766	0.9364
CXCL1	0.008	0.768	0.9364
FGF2	-0.008	0.769	0.9364
CCL28	-0.007	0.775	0.9410
CXCL12	0.007	0.787	0.9449
SH2D1A	0.007	0.796	0.9449
CXADR	0.006	0.805	0.9449
EIF4G1	0.006	0.818	0.9454
ZBTB160	0.006	0.818	0.9454
STAMBP	0.006	0.822	0.9454
IL-7	0.006	0.824	0.9454
CNTNAP2	0.005	0.84	0.9454
CCL4	0.005	0.841	0.9454
EN_RAGE	0.005	0.854	0.9575
Flt3L	0.004	0.877	0.9624
TRIM21	-0.004	0.886	0.9624
EGLN1	0.004	0.89	0.9624
IRAK1	0.004	0.893	0.9624
PLXNA4	-0.003	0.897	0.9624
PRDX5	-0.003	0.905	0.9624
SPRY2	-0.003	0.905	0.9624
SH2B3	-0.003	0.912	0.9630
ADA	-0.002	0.924	0.9647
CST5	0.002	0.942	0.9810
ICA1	-0.001	0.955	0.9851
HEXIM1	-0.001	0.96	0.9851
PRDX1	-0.001	0.971	0.9907
BACH1	-0.001	0.978	0.9921
DFFA	0	0.989	0.9970
MGMT	0	0.996	0.9970

Supplementary Table 6. Association between immune/inflammatory pathway composite scores with EDII scores (partial Spearman correlation's (adjusted for age))

Pathway	rho	p
Extracellular matrix organization	0.116	<.001
Inflammatory response	0.109	<.001
Response to cytokine stimulus	0.082	0.001
MAPK cascade	0.072	0.005
Chemotaxis	0.070	0.006
Regulation of immune response	0.070	0.006
Secretion	0.060	0.019
Cell adhesion	0.055	0.031
Apoptotic process	0.054	0.035
Response to hypoxia	0.027	0.286
Cell activation in immune response	0.008	0.745

Supplementary Table 7. Multivariable logistic regression models of EDII-related proteins with odds of cognitive impairment								
**Primary (Adjusted) Model**								
Protein	Estimate	SE	Z	p	FDR	OR	Lower CI (95%)	Upper CI (95%)
CXCL10	0.198	0.066	2.978	0.003	0.1100	1.219	1.066	1.384
CCL3	0.250	0.087	2.864	0.004	0.1100	1.284	1.086	1.530
ITGA11	-0.362	0.137	-2.636	0.008	0.1467	0.696	0.528	0.905
HGF	0.330	0.139	2.363	0.018	0.2475	1.390	1.054	1.821
OPG	0.353	0.161	2.184	0.029	0.2842	1.423	1.036	1.952
CDCP1	0.224	0.104	2.158	0.031	0.2842	1.251	1.021	1.535
NFATC3	0.208	0.102	2.037	0.042	0.3300	1.231	1.013	1.512
TGF-alpha	0.375	0.193	1.943	0.052	0.3575	1.454	0.995	2.122
IL-12B	0.151	0.089	1.705	0.088	0.5378	1.163	0.982	1.392
IL-10	0.143	0.095	1.496	0.135	0.6875	1.153	0.955	1.390
CLEC4G	-0.239	0.165	-1.446	0.148	0.6875	0.787	0.572	1.095
MMP-10	-0.129	0.092	-1.405	0.160	0.6875	0.879	0.731	1.048
HNMT	0.140	0.105	1.332	0.183	0.6875	1.151	0.942	1.426
MCP-3	0.116	0.088	1.328	0.184	0.6875	1.123	0.942	1.329
CLEC4D	0.122	0.101	1.204	0.229	0.6875	1.129	0.925	1.375
FGF-23	0.131	0.109	1.197	0.231	0.6875	1.140	0.914	1.405
DNER	0.247	0.213	1.156	0.248	0.6875	1.280	0.840	1.941
CKAP4	0.148	0.129	1.148	0.251	0.6875	1.159	0.901	1.492
CCL19	0.069	0.062	1.116	0.264	0.6875	1.072	0.944	1.205
TPSAB1	0.105	0.097	1.092	0.275	0.6875	1.111	0.927	1.355
CD5	-0.153	0.141	-1.087	0.277	0.6875	0.858	0.651	1.132
CD83	0.159	0.151	1.052	0.293	0.6875	1.172	0.871	1.577
CLEC6A	0.112	0.111	1.012	0.312	0.6875	1.119	0.897	1.387
TNF	0.104	0.105	0.992	0.321	0.6875	1.110	0.907	1.370
TREM1	0.132	0.142	0.933	0.351	0.6875	1.142	0.864	1.508
CLEC7A	0.098	0.106	0.926	0.354	0.6875	1.103	0.894	1.354
CSF-1	0.232	0.253	0.916	0.360	0.6875	1.261	0.767	2.069
AREG	0.111	0.124	0.896	0.370	0.6875	1.118	0.868	1.414
IL-18R1	0.118	0.135	0.875	0.382	0.6875	1.125	0.862	1.464
IL-18	0.077	0.091	0.854	0.393	0.6875	1.080	0.908	1.296
CD244	0.078	0.092	0.847	0.397	0.6875	1.082	0.903	1.299
CCL11	0.102	0.121	0.842	0.400	0.6875	1.107	0.872	1.400
TNFRSF9	0.098	0.128	0.765	0.444	0.7296	1.103	0.861	1.424
KLRD1	0.076	0.100	0.753	0.451	0.7296	1.079	0.890	1.320
CCL20	0.034	0.053	0.654	0.513	0.8061	1.035	0.935	1.149
STC1	-0.066	0.111	-0.595	0.552	0.8433	0.936	0.749	1.159
VEGFA	0.060	0.106	0.563	0.573	0.8511	1.062	0.862	1.308

PD-L1	0.062	0.115	0.542	0.588	0.8511	1.064	0.852	1.338
MCP-10	0.061	0.124	0.493	0.622	0.8695	1.063	0.833	1.357
IFNLR1	-0.069	0.152	-0.451	0.652	0.8695	0.934	0.691	1.256
IL-10RB	-0.083	0.185	-0.450	0.653	0.8695	0.920	0.640	1.322
IL-15RA	-0.078	0.179	-0.435	0.664	0.8695	0.925	0.652	1.318
LILRB4	0.040	0.113	0.352	0.725	0.9273	1.040	0.835	1.299
CD8A	0.025	0.080	0.308	0.758	0.9436	1.025	0.877	1.202
CCL23	0.037	0.126	0.290	0.772	0.9436	1.037	0.810	1.331
TRANCE	-0.022	0.103	-0.215	0.830	0.9559	0.978	0.806	1.206
IL-12RB1	-0.024	0.130	-0.186	0.853	0.9559	0.976	0.755	1.259
IL-17A	0.013	0.070	0.184	0.854	0.9559	1.013	0.879	1.159
MASP1	-0.028	0.169	-0.166	0.868	0.9559	0.972	0.699	1.358
IL-6	0.012	0.072	0.164	0.869	0.9559	1.012	0.872	1.157
SIT1	0.011	0.091	0.119	0.905	0.9656	1.011	0.850	1.216
FAM3B	0.014	0.125	0.108	0.914	0.9656	1.014	0.792	1.295
TWEAK	0.009	0.144	0.065	0.948	0.9656	1.009	0.760	1.336
FGF-21	0.004	0.054	0.065	0.948	0.9656	1.004	0.907	1.120
FCRL6	0.000	0.084	0.001	1.000	1.0000	1.000	0.848	1.180
**Unadjusted Model**								
Protein	Estimate	SE	Z	p	FDR	OR	Lower CI (95%)	Upper CI (95%)
CDCP1	0.384	0.097	3.939	<.001	0.0012	1.468	1.214	1.780
ITGA11	-0.421	0.128	-3.294	<.001	0.0028	0.656	0.509	0.842
CXCL10	0.199	0.063	3.178	0.001	0.0138	1.220	1.080	1.380
OPG	0.479	0.151	3.176	0.001	0.0138	1.614	1.203	2.173
HGF	0.356	0.127	2.808	0.005	0.0550	1.427	1.114	1.831
CCL3	0.203	0.079	2.571	0.010	0.0917	1.225	1.051	1.432
TGF-α	0.335	0.170	1.973	0.049	0.3644	1.398	1.003	1.954
IL-12B	0.154	0.079	1.934	0.053	0.3644	1.166	0.998	1.364
NFATC3	0.178	0.097	1.841	0.066	0.4033	1.195	0.989	1.446
AREG	0.191	0.115	1.652	0.098	0.4557	1.210	0.966	1.521
CKAP4	0.187	0.115	1.627	0.104	0.4557	1.205	0.964	1.512
IL-10	0.143	0.090	1.596	0.111	0.4557	1.154	0.969	1.378
HNMT	0.148	0.093	1.583	0.113	0.4557	1.159	0.966	1.393
TREM1	0.196	0.124	1.572	0.116	0.4557	1.216	0.953	1.553
CLEC4G	-0.227	0.152	-1.490	0.136	0.4987	0.797	0.591	1.074
CLEC6A	0.137	0.103	1.322	0.186	0.6394	1.146	0.936	1.405
IL-6	0.084	0.067	1.255	0.210	0.6425	1.088	0.954	1.241
CCL11	0.137	0.115	1.192	0.233	0.6425	1.146	0.916	1.436
TPSAB1	0.107	0.091	1.173	0.241	0.6425	1.113	0.931	1.331
TNFRSF9	0.127	0.111	1.147	0.252	0.6425	1.136	0.914	1.414
TRANCE	-0.109	0.096	-1.136	0.256	0.6425	0.897	0.742	1.082

CCL19	0.066	0.058	1.134	0.257	0.6425	1.068	0.953	1.198
CD83	0.147	0.135	1.087	0.277	0.6443	1.158	0.889	1.510
CD5	-0.137	0.128	-1.068	0.286	0.6443	0.872	0.678	1.121
CCL20	0.052	0.050	1.031	0.303	0.6443	1.053	0.955	1.162
KLRD1	0.092	0.093	0.982	0.326	0.6443	1.096	0.913	1.317
CSF-1	0.219	0.225	0.970	0.332	0.6443	1.244	0.800	1.938
MCP-3	0.078	0.081	0.960	0.337	0.6443	1.081	0.922	1.268
IL-18R1	0.116	0.122	0.954	0.340	0.6443	1.123	0.885	1.426
CLEC4D	0.085	0.092	0.925	0.355	0.6443	1.089	0.910	1.304
MMP-10	-0.076	0.084	-0.901	0.368	0.6443	0.927	0.786	1.093
TNF	0.084	0.098	0.861	0.389	0.6443	1.088	0.899	1.319
LILRB4	0.082	0.099	0.832	0.405	0.6443	1.086	0.895	1.319
CLEC7A	0.079	0.096	0.824	0.410	0.6443	1.082	0.897	1.307
TWEAK	-0.111	0.134	-0.824	0.410	0.6443	0.895	0.688	1.165
IFNLR1	-0.107	0.141	-0.758	0.448	0.6844	0.898	0.681	1.185
IL-18	0.055	0.085	0.651	0.515	0.7570	1.057	0.895	1.248
IL-17A	0.043	0.067	0.639	0.523	0.7570	1.044	0.916	1.190
FGF-23	0.059	0.098	0.605	0.545	0.7686	1.061	0.876	1.287
FGF-21	0.027	0.049	0.545	0.586	0.8058	1.027	0.932	1.132
FCRL6	0.033	0.079	0.417	0.677	0.8960	1.034	0.885	1.208
CD244	0.030	0.088	0.339	0.734	0.8960	1.030	0.868	1.224
MCP-10	0.038	0.116	0.329	0.742	0.8960	1.039	0.828	1.305
PD-L1	0.032	0.107	0.300	0.764	0.8960	1.033	0.837	1.274
MASP1	-0.047	0.156	-0.299	0.765	0.8960	0.954	0.702	1.297
FAM3B	0.034	0.113	0.298	0.766	0.8960	1.034	0.829	1.290
DNER	0.057	0.194	0.292	0.770	0.8960	1.058	0.723	1.549
IL12RB1	0.033	0.120	0.277	0.782	0.8960	1.034	0.817	1.310
IL-10RB	-0.034	0.164	-0.208	0.835	0.9372	0.967	0.701	1.333
IL-15RA	-0.024	0.161	-0.152	0.880	0.9533	0.976	0.712	1.339
SIT1	0.012	0.085	0.146	0.884	0.9533	1.012	0.858	1.195
STC1	-0.007	0.103	-0.070	0.944	0.9869	0.993	0.811	1.215
CD8A	-0.005	0.074	-0.062	0.951	0.9869	0.995	0.860	1.152
VEGFA	0.002	0.098	0.016	0.987	0.9870	1.002	0.827	1.214
CCL23	0.002	0.116	0.016	0.987	0.9870	1.002	0.798	1.258
**Sensitivity Analyses Lifestyle Model**								
Protein	Estimate	SE	Z	p	FDR	OR	Lower CI (95%)	Upper CI (95%)
ITGA11	-0.415	0.142	-2.931	0.003	0.1100	0.660	0.499	0.870
CXCL10	0.195	0.068	2.847	0.004	0.1100	1.215	1.063	1.391
CCL3	0.248	0.090	2.751	0.006	0.1100	1.281	1.076	1.533
NFATC3	0.244	0.105	2.317	0.021	0.2292	1.276	1.039	1.572

CDCP1	0.247	0.107	2.301	0.021	0.2292	1.280	1.038	1.581
HGF	0.324	0.144	2.247	0.025	0.2292	1.383	1.043	1.837
OPG	0.333	0.165	2.016	0.044	0.3457	1.396	1.010	1.932
TGF-α	0.369	0.202	1.830	0.067	0.4606	1.446	0.976	2.152
IL-10	0.160	0.098	1.632	0.103	0.5665	1.174	0.969	1.425
IL-12B	0.151	0.093	1.630	0.103	0.5665	1.163	0.970	1.396
CLEC4D	0.166	0.105	1.576	0.115	0.5750	1.181	0.961	1.453
TREM1	0.224	0.151	1.482	0.138	0.5971	1.251	0.931	1.684
MMP-10	-0.136	0.094	-1.449	0.147	0.5971	0.872	0.725	1.049
MCP-3	0.129	0.090	1.433	0.152	0.5971	1.138	0.954	1.358
HNMT	0.150	0.109	1.377	0.169	0.6197	1.162	0.939	1.441
CCL19	0.084	0.065	1.294	0.196	0.6215	1.087	0.958	1.234
CD83	0.199	0.157	1.266	0.206	0.6215	1.220	0.897	1.661
CLEC6A	0.147	0.116	1.264	0.206	0.6215	1.158	0.923	1.455
TNF	0.130	0.107	1.212	0.225	0.6215	1.139	0.924	1.407
CLEC4G	-0.208	0.172	-1.210	0.226	0.6215	0.812	0.579	1.137
FGF-23	0.124	0.113	1.096	0.273	0.7050	1.132	0.908	1.417
CCL11	0.135	0.125	1.076	0.282	0.7050	1.144	0.895	1.464
TPSAB1	0.104	0.100	1.039	0.299	0.7150	1.110	0.912	1.351
CCL20	0.054	0.055	0.989	0.323	0.7272	1.056	0.948	1.175
CD5	-0.138	0.145	-0.947	0.344	0.7272	0.871	0.655	1.159
AREG	0.121	0.129	0.936	0.349	0.7272	1.128	0.877	1.455
DNER	0.203	0.221	0.920	0.357	0.7272	1.225	0.795	1.890
CKAP4	0.106	0.133	0.799	0.424	0.7629	1.112	0.858	1.446
CD244	0.076	0.095	0.796	0.426	0.7629	1.079	0.895	1.301
KLRD1	0.082	0.104	0.792	0.428	0.7629	1.086	0.886	1.332
IL-18	0.073	0.093	0.789	0.430	0.7629	1.076	0.897	1.292
STC1	-0.082	0.115	-0.709	0.478	0.7749	0.922	0.735	1.154
CSF-1	0.185	0.262	0.709	0.479	0.7749	1.204	0.721	2.011
IL-15RA	-0.131	0.185	-0.708	0.479	0.7749	0.877	0.610	1.261
CLEC7A	0.074	0.110	0.672	0.502	0.7878	1.077	0.868	1.336
TNFRSF9	0.085	0.132	0.640	0.522	0.7878	1.088	0.840	1.411
MCP-10	0.081	0.129	0.629	0.530	0.7878	1.085	0.842	1.399
VEGFA	0.062	0.109	0.564	0.573	0.8293	1.064	0.859	1.318
IL-18R1	0.073	0.142	0.517	0.605	0.8532	1.076	0.815	1.421
PD-L1	0.059	0.119	0.495	0.621	0.8539	1.061	0.840	1.340
FGF-21	0.026	0.056	0.469	0.639	0.8572	1.026	0.920	1.145
IL-6	0.032	0.075	0.433	0.665	0.8708	1.033	0.892	1.196
LILRB4	0.047	0.116	0.403	0.687	0.8750	1.048	0.834	1.317

IL-10RB	-0.073	0.190	-0.385	0.700	0.8750	0.929	0.640	1.349
SIT1	0.029	0.095	0.303	0.762	0.9303	1.029	0.854	1.241
IL-17A	0.019	0.072	0.263	0.792	0.9303	1.019	0.885	1.175
IFNLR1	-0.041	0.156	-0.260	0.795	0.9303	0.960	0.707	1.304
IL12RB1	-0.030	0.137	-0.223	0.823	0.9361	0.970	0.742	1.269
CCL23	0.027	0.131	0.209	0.834	0.9361	1.028	0.795	1.328
MASP1	-0.030	0.174	-0.173	0.862	0.9371	0.970	0.690	1.364
TWEAK	-0.024	0.149	-0.164	0.870	0.9371	0.976	0.729	1.307
FAM3B	-0.016	0.129	-0.128	0.898	0.9371	0.984	0.764	1.266
FCRL6	-0.011	0.087	-0.122	0.903	0.9371	0.989	0.833	1.174
TRANCE	0.006	0.106	0.059	0.953	0.9560	1.006	0.818	1.238
CD8A	0.005	0.083	0.055	0.956	0.9560	1.005	0.854	1.181

Adjusted results derived from multivariable linear regression models adjusted for age, geographical region, hormone treatment, education, *APOE* ε4 status, diabetes, obesity, hypertension, eGFR-creatinine. Sensitivity analyses were additionally adjusted for lifestyle factors (i.e., alcohol intake, smoking status, physical activity, and sleep quality).

Supplementary Table 8. Biological process, molecular functions, and diseases associated with each inflammatroy diet candidate protein.

Gene	Gene Ontology[A]	Disease[B]	Solr Score[C]	PMID[D]
CDCP1	*Molecular Processes*	Pancreatic Cancer (PNC035)	4.11	· 32341034
	· protein binding			· 33060289
				· 30396925
				· 24384474
				· 20501830
		Prostate Cancer (PRS040)	2.929	· 19916495
				· 19671673
				· 22390300
				· 32250342
				· 22457534
		Renal Cell Carcinoma, Nonpapillary (RNL114)	2.729	· 18483744
				· 21233420
				· 23378636
CCL3	*Biological Processes*	Myeloma Multiple (MYL069)	4.099	· 16263571
	· MAPK cascade			· 10887133
	· osteoblast differentiation			· 14510950
	· cell activation			· 12492576
	· monocyte chemotaxis			· 12506012
	· calcium ion transport			
	· cellular calcium ion homeostasis			
	· exocytosis			
	· chemotaxis			
	· inflammatory response			
	· cytoskeleton organization			
	· G protein-coupled receptor signaling pathway			
	· cell-cell signaling			
	· regulation of cell shape			
	· response to toxic substance			
	· positive regulation of gene expression			
	· negative regulation of gene expression			
	· T cell chemotaxis			
	· release of sequestered calcium ion into cytosol by sarcoplasmic reticulum			
	· calcium mediated signaling			

	· signaling			
	· positive regulation of cell migration			
	· negative regulation of bone mineralization			
	· neutrophil chemotaxis			
	· lipopolysaccharide-mediated signaling pathway			
	· eosinophil degranulation			
	· protein kinase B signaling			
	· positive regulation of GTPase activity			
	· negative regulation by host of viral transcription			
	· negative regulation of osteoclast differentiation			
	· eosinophil chemotaxis			
	· lymphocyte chemotaxis			
	· regulation of behavior			
	· positive regulation of calcium-mediated signaling			
	· positive regulation of protein kinase B signaling			
	· positive regulation of calcium ion transport			
	· regulation f sensory perception of pain cell chemotaxis			
	· chemokine-mediated signaling pathway			
	· positive regulation of ERK1 and ERK2 cascade			
	· response to cholesterol			
	· cellular response to interferon-gamma			
	· cellular response to interleukin-1			
	· cellular response to tumor necrosis factor			
	· cellular response to organic cyclic compound			
	· granulocyte chemotaxis			
	· positive regulation of calcium ion import			
	· positive regulation of microglial cell activation			
	· positive regulation of microglial cell migration			
	· positive regulation of natural killer cell chemotaxis			
	Molecular Functions			
	· protein kinase activity			

	· calcium-dependent protein kinase C activity			
	· protein binding			
	· chemokine activity			
	· phospholipase activator activity			
	· kinase activity			
	· CCR1 chemokine receptor binding			
	· CCR5 chemokine receptor binding			
	· chemoattractant activity			
	· identical protein binding	Leukemia (LKM002)	3.718	· 9546433
	· CCR chemokine receptor binding			· 7492787
				· 19279110
				· 9371282
		Myeloid Leukemia (MYL006)	3.718	· 8265663
				· 7769832
				· 9546433
				· 9669675
				· 8843546
CXCL10	*Biological Processes*	Non-Alcoholic Steatohepatitis (NNL006)	2.989	· 27669973
	· chemotaxis			· 28824718
	· inflammatory response			· 30964207
	· signal transduction			· 28262979
	· cell surface receptor signaling pathway			
	· G protein-coupled receptor signaling pathway			
	· adenylate cyclase-activating G protein-coupled receptor signaling pathway			
	· cell-cell signaling			
	· muscle organ development			
	· blood circulation			
	· T cell chemotaxis			
	· regulation of T cell chemotaxis			
	· negative regulation of angiogenesis			
	· neutrophil-chemotaxis			
	· endothelial cell activation			
	· regulation of cell population proliferation			
	· regulation of apoptotic process			
	· positive regulation of transcription by RNA polymerase II			

	· positive regulation of release of sequestered calcium ion into cytosol			
	· antimicrobial humoral immune response mediated by antimicrobial peptide			
	· chemokine-mediated signaling pathway			
	· cellular response to lipopolysaccharide			
	· positive regulation of monocyte chemotaxis			
	· cellular response to virus			
	· antiviral innate immune response			
	· regulation of endothelial tube morphogenesis			
	· positive regulation of T cell migration			
	Molecular Functions			
	· signaling receptor binding			
	· protein binding			
	· chemokine activity			
	· heparin binding			
	· cAMP-dependent protein kinase regulator activity			
	· chemoattractant activity			
	· CXCR chemokine receptor binding	Non-Alcoholic Fatty Liver Disease (NNL005)	2.911	· 27669973
	· CXCR3 chemokine receptor binding			· 28824718
				· 30964207
		Fatty Liver Disease (FTT001)	2.911	· 27669973
				· 28824718
				· 30964207
HGF	*Biological Processes*	Liver Disease (LVR013)	12.84	· 14696449
	· mitotic cell cycle			· 1824837
	· epithelial to mesenchymal transition			· 11995475
	· liver development			· 8778194
	· positive regulation of protein phosphorylation			· 11264569
	· proteolysis			
	· negative regulation of autophagy			
	· positive regulation of phosphatidylinositol 3-kinase signaling			
	· positive regulation of cell migration			
	· cellular response to hepatocyte growth factor stimulus			
	· negative regulation of apoptotic process			

	· negative regulation of cysteine-type endopeptidase activity involved in apoptotic process			
	· positive regulation of osteoblast differentiation			
	· positive regulation of transcription by RNA polymerase II			
	· hepatocyte growth factor receptor signaling pathway			
	· positive regulation of peptidyl-tyrosine phosphorylation			
	· cell chemotaxis			
	· regulation of branching involved in salivary gland morphogenesis by mesenchymal-epithelial signaling			
	· negative regulation of release of cytochrome c from mitochondria			
	· negative regulation of hydrogen peroxide-mediated programmed cell death			
	· positive regulation of DNA biosynthetic process			
	Molecular Functions			
	· endopeptidase activity			
	· serine-type endopeptidase activity			
	· signaling receptor binding			
	· protein binding			
	· growth factor activity			
	· chemoattractant activity			
	· identical protein binding	Acute Liver Disease (ACT134)	10.925	· 1824837
				· 1530786
				· 7600833
				· 9286233
				· 8175137
		Hepatocellular Carcinoma (HPT023)	10.344	· 12527708
				· 16627020
				· 10190737
				· 11264569
				· 8778194
NFATC3	*Biological Processes*	Hypoxia (HYP266)	1.84	· 32188205
	· cytokine production			
	· regulation of transcription by RNA polymerase II			

	· inflammatory response			
	· calcineurin-NFAT signaling cascade			
	· positive regulation of transcription by RNA polymerase II			
	· negative regulation of pri-miRNA transcription by RNA polymerase II			
	· negative regulation of vascular associated smooth muscle cell differentiation			
	Molecular Functions			
	· RNA polumerase II cis-regulatory region sequence-specific DNA binding			
	· DNA-binding transcription repressor activity, RNA polymerase II-binding			
	· DNA binding transcription activator activity, RNA polymerase II-specific			
	· protein binding			
	· transcription factor binding	Myocardial Infarction (MYC007)	0.271	· 32010259
	· sequence-specific double-stranded DNA binding	Tooth Agenesis (TTH002)	0.271	· 28265457
ITGA11	*Biological Processes*	Lung Cancer (LNG032)	1.061	· 17016581
	· osteoblast differentiation			
	· substrate-dependent cell migration			
	· cell adhesion			
	· cell-matrix adhesion			
	· integrin-mediated signaling pathway			
	· muscle organ development			
	· cell adhesion mediated by integrin			
	· collagen-activated signaling pathway			
	Molecular Functions			
	· collagen binding			
	· collagen receptor activity	Lung Cancer Susceptibility 3 (LNG064)	1.061	· 12386823
	· collagen binding involved in cell-matrix adhesion	Tick Infestation (TCK004)	1.061	· 20565915
OPG	*Biological Processes*	Bone Reabsorption	5.02	· 10829073
	· skeletal system development			· 15860239

	Molecular Functions	Disease (BNR002)		· 11594776
	· cytokine activity			· 9790989
	· protein binding			· 10234572
	· signaling receptor activity			
		Osteoporosis (OST002)	3.256	· 15632471
				· 15003790
				· 9647741
				· 10234572
				· 11594776
		Bone Mineral Density Quantitative Trait Locus 15 (BNM029)	2.912	· 10234572
				· 15632471
				· 11594776
				· 15003790
				· 9647741
A. GeneCards Gene Ontology (GO) terms for Biological Processes and/or Molecular Functions supported by peer-reviewed documentation B. Disease classification in MalaCard Human Disease Database (MalaCard ID) C. Solr score of gene-disease annotations* D. PubMed IDs of gene-disease associated literature via MalaCard. * Calculated from the Apache Lucene platform by integrating, indexing and clustering data to indicate scored diseases whose annotations contain the search string of the Gene (Rappaport et al., 2013; Rappaport et al., 2017).				
Referneces: Rappaport, N., Nativ, N., Stelzer, G., Twik, M., Guan-Golan, Y., Stein, T. I., . . . Lancet, D. (2013). MalaCards: an integrated compendium for diseases and their annotation. Database (Oxford), 2013, bat018. doi:10.1093/database/bat018; Rappaport, N., Twik, M., Plaschkes, I., Nudel, R., Iny Stein, T., Levitt, J., . . . Lancet, D. (2017). MalaCards: an amalgamated human disease compendium with diverse clinical and genetic annotation and structured search. Nucleic Acids Res, 45(D1), D877-d887. doi:10.1093/nar/gkw1012				

Supplementary Table 9. Multivariable logistic regression models of EDII-related immune/inflammatory pathways with odds of cognitive impairment

Pathway	Estimate	SE	Z	p	OR	Lower CI (95%)	Upper CI (95%)
**Primary (Adjusted) Model**							
Pathway	Estimate	SE	Z	p	OR	Lower CI (95%)	Upper CI (95%)
Apoptotic process	0.113	0.055	2.029	0.042	1.119	1.004	1.248
Regulation of immune response	0.107	0.056	1.907	0.057	1.112	0.997	1.242
Response to cytokine stimulus	0.107	0.056	1.895	0.058	1.113	0.997	1.243
Secretion	0.104	0.056	1.866	0.062	1.110	0.995	1.238
MAPK cascade	0.096	0.056	1.713	0.087	1.100	0.987	1.228
Cell adhesion	0.087	0.056	1.552	0.121	1.091	0.978	1.219
Inflammatory response	0.088	0.057	1.549	0.121	1.092	0.977	1.222
Chemotaxis	0.078	0.056	1.410	0.158	1.081	0.970	1.206
Extracellular matrix organization	0.035	0.056	0.628	0.530	1.036	0.929	1.155
**Unadjusted Model**							
Pathway	Estimate	SE	Z	p	OR	Lower CI (95%)	Upper CI (95%)
Apoptotic process	0.096	0.051	1.873	0.061	1.101	0.996	1.218
Secretion	0.096	0.052	1.869	0.062	1.101	0.996	1.218
Regulation of immune response	0.093	0.051	1.811	0.070	1.098	0.993	1.215
Response to cytokine stimulus	0.092	0.051	1.785	0.074	1.096	0.991	1.213
Inflammatory response	0.083	0.051	1.619	0.106	1.087	0.983	1.203
MAPK cascade	0.077	0.051	1.502	0.133	1.080	0.977	1.195
Cell adhesion	0.068	0.051	1.325	0.185	1.071	0.968	1.184
Chemotaxis	0.067	0.051	1.298	0.194	1.069	0.967	1.183
Extracellular matrix organization	0.067	0.052	1.297	0.195	1.069	0.967	1.183

Adjusted results derived from multivariable linear regression models adjusted for age, geographical region, hormone treatment, education, *APOE* ε4 status, diabetes, obesity, hypertension, eGFR-creatinine.

**Supplementary Table 10.** Fixed effect meta-analysis examining candidate proteins using cohort specific effect estimates WHIMS and ESTHER					
Protein	Cohort	Odds Ratio	95%CI-Lower	95%CI-Upper	p
CCL3	WHIMS	1.284	1.086	1.530	0.004
	ESTHER	1.140	1.020	1.270	0.026
	Both	1.161	1.096	1.226	< 0.001
CXCL10	WHIMS	1.219	1.066	1.384	0.003
	ESTHER	1.160	1.030	1.300	0.014
	Both	1.166	1.124	1.208	< 0.001
CDCP1	WHIMS	1.251	1.021	1.535	0.031
	ESTHER	1.200	1.060	1.360	0.003
	Both	1.202	1.161	1.243	< 0.001
HGF	WHIMS	1.390	1.054	1.821	0.018
	ESTHER	1.340	1.180	1.520	< 0.001
	Both	1.341	1.308	1.374	< 0.001
OPG	WHIMS	1.423	1.036	1.952	0.029
	ESTHER	1.390	1.220	1.580	< 0.001
	Both	1.390	1.362	1.418	< 0.001
*Two plasma proteins associated with cognitive impairment in WHIMS (NFATC3, ITGA11) were not measured in ESTHER. WHIMS participants met criteria for MCI/dementia; ESTHER participants met criteria for dementia					
Results derived from multivariable logistic regression models adjusted for covariates used in WHIMS (age, APOE ε4 status, diabetes, education, obesity, hypertension, eGFR- creatinine, geographical region, hormone treatment) and in ESTHER (age, APOE ε4 status, diabetes, education, BMI, cardiovascular disease, depression, physical activity, sex)					

**Supplementary Table 11.** ARIC participant characteristics stratified according to late-life baseline (2011-2013) cognitive status			
Characteristic, n (%) or mean (SD)	Full sample	Cognitively normal at baseline	Mild cognitive impairment at baseline
	n = 4,288	n = 3,383	n = 905
Demographic variables			
Age, mean (SD)	75.2 (5.0)	74.8 (4.8)	76.5 (5.2)
Men, No. (%)	1,806 (42.1)	1,364 (40.3)	442 (48.8)
Women, No. (%)	2,482 (57.9)	2,019 (59.7)	463 (51.2)
Black, No. (%)	780 (18.2)	618 (18.3)	162 (17.9)
White, No. (%)	3,508 (81.8)	2,765 (81.7)	743 (82.1)
Center, No. (%)			
Minneapolis, Minnesota	1,388 (32.4)	1,115 (33.0)	273 (30.2)
Washington County, Maryland	1,197 (27.9)	931 (27.5)	266 (29.4)
Forsyth Count, North Carolina	981 (22.9)	769 (22.7)	212 (23.4)
Jackson, Mississippi	722 (16.8)	568 (16.8)	154 (17.0)
Education, No. (%)			
Less than high school	512 (11.9)	399 (11.8)	113 (12.5)
High school/GED/vocational	1,831 (42.7)	1,414 (41.8)	417 (46.1)
College (at least >1 year)	1,945 (45.4)	1,570 (46.4)	375 (41.4)
Apolipoprotein E ε4 alleles, No. (%)			
0	3,002 (70.0)	2,402 (71.0)	600 (66.3)
≥ 1	1,152 (26.9)	872 (25.8)	280 (30.9)
Missing	134 (3.1)	109 (3.2)	25 (2.8)
Physiological and lab variables, mean (SD)			
Body mass index, kg/m2	28.8 (5.6)	28.7 (5.6)	28.8 (5.6)
Total cholesterol, mg/dl	181.5 (40.9)	178.2 (41.8)	182.4 (40.6)

eGFR, mean, (SD)	70.1 (16.7)	70.7 (16.5)	68.1 (17.6)
Cardiovascular risk factors, No. (%)			
Hypertension	3,137 (73.2)	2,446 (72.3)	691 (76.4)
Diabetes mellitus	1,186 (27.7)	885 (26.2)	301 (33.3)
Coronary heart disease	631 (15.0)	472 (14.2)	159 (17.9)
Cigarette smoking, current	247 (5.8)	198 (5.9)	49 (5.4)
Cognitive status, No. (%)			
Cognitively normal	3,383 (78.9)	--	--
Mild cognitive impairment	905 (21.1)	--	--

Supplementary Table 12. Correlations of candidate protein measurements across Olink® and Somscan® platforms (Spearman's)

Protein target	SomaScan SeqID	EntrezGeneSymbol	Olink varname	OlinkID	rho (Pietzner et al., (2021))	rho (ARIC)
IP-10	SeqId_4141_79	CXCL10	CXCL10_INF	OID00535	0.723	0.842
MIP-1a	SeqId_3040_59	CCL3	CCL3_CAR2	OID00440	0.091	*
CDCP1	SeqId_16818_200	CDCP1	CDCP1_INF	OID00476	0.698	0.832
HGF	SeqId_2681_23	HGF	HGF_INF	OID00522	0.413	0.529
OPG	SeqId_8304_50	TNFRSF11B	OPG_CAR3	OID00571	0.577	*

Reference: Pietzner, M. et al., (2021). doi.org/10.1038/s41467-021-27164-0.

*Not measured on Olink; only measured on Somascan in ARIC

Supplementary Table 13. Association of inflammatory diet proteins with time-to-dementia onset in ARIC

Protein	Cohort	Odds Ratio	95%CI-Lower	95%CI-Upper	p
CCL3	Men	0.977	0.768	1.243	0.851
	Women	1.198	0.973	1.475	0.089
	Full sample	1.087	0.931	1.270	0.290
CXCL10	Men	0.958	0.782	1.175	0.683
	Women	1.106	0.928	1.319	0.261
	Full sample	1.034	0.906	1.181	0.616
CDCP1	Men	1.339	1.094	1.639	0.005
	Women	1.224	1.033	1.449	0.019
	Full sample	1.264	1.112	1.438	< 0.001
HGF	Men	1.256	1.033	1.527	0.022
	Women	1.237	1.022	1.496	0.029
	Full sample	1.245	1.087	1.425	0.002
OPG	Men	1.600	1.173	2.183	0.003
	Women	1.538	1.186	1.994	0.001
	Full sample	1.516	1.243	1.849	< 0.001

*Two plasma proteins associated with cognitive impairment in WHIMS (NFATC3, ITGA11) were not measured in ARIC. WHIMS participants met criteria for MCI/dementia; ARIC participants met criteria for dementia.

Results derived from Cox proportional hazard regression models adjusted for age, center-race, sex, education, *APOE* ε4 status, diabetes, BMI, smoking status, hypertension, and eGFR-creatinine.

Supplementary Table 14. Correlations of variables related to EDII as well as cognitive impairment with plasma biomarkers of AD pathology (AB42/40) and neurodegeneration (NfL) (partial Spearman correlation's (adjusted for age))

$Ab_{42/40}$	rho	p	NfL	rho	p
HGF	0.116	<.001	OPG	0.120	<.001
NFATC3	0.138	<.001	CCL3	0.079	0.003
CCL3	0.119	<.001	CXCL10	0.060	0.020
OPG	0.082	0.002	CDCP1	0.053	0.043
CXCL10	0.052	0.045	ITGA11	0.047	0.076
ITGA11	0.048	0.067	NFATC3	-0.043	0.101
CDCP1	0.027	0.295	HGF	0.002	0.943
Apoptotic process	0.194	<.001	Apoptotic process	0.098	<.001

**Supplementary Table 15.** BLSA participant characteristics stratified according to low and high SPARE-AD groups (median split)			
Characteristic	Total Sample	Low SPARE-AD	High SPARE-AD
	n = 970	n = 485	n = 485
SPARE-AD Score	-1.3 (0.9)	-2.0 (0.5)	-0.7 (0.6)
Demographic Variables, No. (%)			
Age (years) mean, (SD)	66.0 (14.8)	60.5 (14.5)	71.5 (13.0)
Female	534 (55.1)	266 (54.8)	268 (55.4)
White Race	639 (66.0)	325 (67.1)	314 (64.9)
Non-White Race	329 (34.0)	159 (32.9)	170 (35.1)
Education (years) mean, (SD)	17.1 (2.4)	17.3 (2.3)	16.9 (2.6)
APOEε4 alleles, No. (%)			
0 ε4 alleles	636 (65.6)	302 (62.3)	334 (68.9)
1-2 ε4 alleles	240 (24.7)	122 (25.2)	118 (24.3)
Missing	94 (9.7)	61 (12.6)	33 (6.8)
Comorbidity variables, No. (%)			
Hypertension	319 (33.1)	127 (26.3)	192 (39.8)
Diabetes mellitus	51 (5.3)	28 (5.8)	23 (4.8)
Obesity	213 (22.0)	110 (22.7)	103 (21.4)
Heart ischemic disease	56 (5.8)	18 (3.7)	38 (7.9)
Congestive heart failure	64 (6.6)	20 (4.1)	44 (9.1)
Cancer	97 (10.1)	43 (9.0)	54 (11.2)
COPD	121 (12.5)	49 (10.1)	72 (14.9)
CKD	185 (19.1)	58 (12.0)	127 (26.3)
eGFR, mean, (SD)	82.5 (17.4)	86.6 (16.7)	78.5 (17.1)
Cognitive status, No. (%)			
Cognitively normal	870 (89.7)	460 (94.8)	410 (84.5)
Impaired but not MCI	13 (1.3)	6 (1.2)	7 (1.4)
MCI	47 (4.8)	15 (3.1)	32 (6.6)
Dementia	40 (4.1)	4 (0.0)	36 (7.4)

**Supplementary Table 16.** Associations of inflammatory diet proteins with the SPARE-AD in the BLSA							
Protein	SomaScan SeqID	Beta Estimate	SE	*t* value	p	Lower CI (95%)	Upper CI (95%)
CXCL10	SeqId_4141_79	-0.031	0.053	-0.589	0.556	0.874	1.075
CCL3	SeqId_3040_59	0.194	0.063	3.095	0.002	1.074	1.373
CDCP1	SeqId_16818_200	0.152	0.055	2.742	0.006	1.044	1.297
HGF	SeqId_2681_23	0.092	0.064	1.442	0.150	0.967	1.243
OPG	SeqId_8304_50	0.239	0.069	3.441	0.001	1.108	1.455
ITGA11	SeqId_20181-17	0.108	0.102	1.061	0.289	0.912	1.360
* NFATC3 not measured in BLSA							
Results derived from multivariable linear regression models adjusted for age, sex, race, education, APOEε4, eGFR-creatinine and a comorbidity index, calculated as the total percentage of eight comorbid conditions: obesity, hypertension, diabetes, cancer, ischemic heart disease, chronic heart failure, chronic kidney disease and chronic obstructive pulmonary disease.							

**Supplementary Table 17.** Consensus transcript expression levels (normalized Transcripts per Million; nTPM) of genes encoding for candidate proteins across immune tissues and cells of interest						
Gene	Immune Tissue Type	nTPM		Gene	Immune Cell Type	nTPM
CCL3	tonsil	0.00		CCL3	T-cells	248.00
CCL3	thymus	0.00		CCL3	NK-cells	106.10
CCL3	spleen	49.80		CCL3	monocytes	23.50
CCL3	lymph node	1.80		CCL3	granulocytes	23.50
CCL3	bone marrow	77.50		CCL3	dendritic cells	441.40
CCL3	appendix	8.20		CCL3	B-cells	45.30
CDCP1	tonsil	5.90		CDCP1	T-cells	1.80
CDCP1	thymus	0.60		CDCP1	NK-cells	0.00
CDCP1	spleen	0.70		CDCP1	monocytes	10.70
CDCP1	lymph node	0.80		CDCP1	granulocytes	8.80
CDCP1	bone marrow	0.90		CDCP1	dendritic cells	0.30
CDCP1	appendix	4.40		CDCP1	B-cells	0.30
CXCL10	tonsil	17.50		CXCL10	T-cells	1.80
CXCL10	thymus	35.60		CXCL10	NK-cells	0.00
CXCL10	spleen	20.50		CXCL10	monocytes	13.50
CXCL10	lymph node	68.50		CXCL10	granulocytes	1.30
CXCL10	bone marrow	1.20		CXCL10	dendritic cells	0.00
CXCL10	appendix	96.40		CXCL10	B-cells	2.20
HGF	tonsil	1.50		HGF	T-cells	0.60
HGF	thymus	1.50		HGF	NK-cells	1.00
HGF	spleen	6.50		HGF	monocytes	10.50
HGF	lymph node	2.20		HGF	granulocytes	1.30
HGF	bone marrow	12.40		HGF	dendritic cells	0.00
HGF	appendix	6.40		HGF	B-cells	0.00
ITGA11	tonsil	0.10		ITGA11	T-cells	0.60
ITGA11	thymus	0.20		ITGA11	NK-cells	0.00
ITGA11	spleen	3.20		ITGA11	monocytes	0.00
ITGA11	lymph node	0.50		ITGA11	granulocytes	0.00
ITGA11	bone marrow	0.20		ITGA11	dendritic cells	0.30
ITGA11	appendix	1.10		ITGA11	B-cells	0.30
NFATC3	tonsil	22.90		NFATC3	T-cells	59.40
NFATC3	thymus	103.00		NFATC3	NK-cells	129.80
NFATC3	spleen	17.80		NFATC3	monocytes	40.60
NFATC3	lymph node	23.50		NFATC3	granulocytes	49.40
NFATC3	bone marrow	23.20		NFATC3	dendritic cells	64.90
NFATC3	appendix	12.10		NFATC3	B-cells	41.80
OPG	tonsil	2.90		OPG	T-cells	0.40
OPG	thymus	1.30		OPG	NK-cells	0.00

OPG	spleen	2.60		OPG	monocytes	8.30
OPG	lymph node	7.10		OPG	granulocytes	0.00
OPG	bone marrow	0.00		OPG	dendritic cells	0.20
OPG	appendix	5.40		OPG	B-cells	0.70

**Supplementary Table 18.** Differential expression of genes encoding for candidate proteins (measured via RNA-seq) following *in vitro* immune challenge (LPS Treatment)									
	Author								
Gene	Alasso et al., *	Das et al., **	Pinilla-Vera et al., *	Bush et al., **	Buscher et al., *	Lissner et al., *	Buerfent et al., *	Bennett et al., *	Jager et al. *
CCL3	UP	UP	UP	UP	-	UP	UP	UP	UP
CXCL10	UP	UP	UP	UP	UP	UP	-	UP	UP
CDCP1	-	-	-	-	-	-	-	-	DOWN
HGF	-	-	DOWN	-	-	-	DOWN	-	-
OPG	-	-	-	-	-	-	-	-	-
ITGA11	-	-	-	-	-	-	-	-	-
NFATC3	DOWN	-	DOWN	-	DOWN	-	DOWN	-	-
*Significance defined as reported adjusted p values (<.05).									
**Significance defined as >3 Fold Change									

**Author**	**Cell Type**
Alasso et al.,	Macrophages (iPSC & PBMC derived; Human)
Das et al.,	Macrophages (Bone Marrow derived; Mice; Adult)
Pinilla-Vera et al.,	Macrophages (Primary Alveolar; Human)
Bush et al.,	Macrophages (Bone Marrow derived; Buffalo, Cow, Horse, Mouse, Rat; Adult)
Buscher et al.,	Macrophages (Peritoneal derived; Mouse;Adult)
Lissner et al.,	Monocytes (Umbilical cord derived; Human)
Buerfent et al.,	Monocytes (PBMC derived; Human)
Bennett et al.,	Microglia (Brain, Cell sorted;Mice; Adult)
Jager et al.,	Dendritic Cells (Bone Marrow derived; Mouse;Adult)

Author	Title
Alasso et al.,	Transcriptional profiling of macrophages derived from monocytes and iPS cells identifies a conserved response to LPS and novel alternative transcription
Das et al.,	High-Resolution Mapping and Dynamics of the Transcriptome, Transcription Factors, and Transcription Co-Factor Networks in Classically and Alternatively Activated Macrophages
Pinilla-Vera et al.,	Full Spectrum of LPS Activation in Alveolar Macrophages of Healthy Volunteers by Whole Transcriptomic Profiling

Bush et al.,	Species-Specificity of Transcriptional Regulation and the Response to Lipopolysaccharide in Mammalian Macrophages
Buscher et al.,	Natural variation of macrophage activation as disease-relevant phenotype predictive of inflammation and cancer survival
Lissner et al.,	Age-Related Gene Expression Differences in Monocytes from Human Neonates, Young Adults, and Older Adults
Buerfent et al.,	Transcriptome-wide analysis of filarial extract-primed human monocytes reveal changes in LPS-induced PTX3 expression levels
Bennett et al.,	New tools for studying microglia in the mouse and human CNS
Jager et al.,	Dendritic Cells Regulate GPR34 through Mitogenic Signals and Undergo Apoptosis in Its Absence

Supplementary Table 19. Gene Enrichment analyses using PANTHER functional classification platform					
Analysis Type: PANTHER Overrepresentation Test (Released 20210224)					
Annotation Version and Release Date: GO Ontology database DOI: 10.5281/zenodo.5725227 Released 2021-11-16					
Reference List: Homo sapiens (all genes in database)					
Correction: FDR					
GO biological process	Homo sapiens - REFLIST (20595)	# Proteins Identified in Pathway	raw P-value	FDR	Proteins
positive chemotaxis (GO:0050918)	45	3	0.000000413	0.006470000	CXCL10, CCL3, HGF
GO molecular function	Homo sapiens - REFLIST (20595)	# Proteins Identified in Pathway	raw P-value	FDR	Proteins
chemoattractant activity (GO:0042056)	35	3	0.000000202	0.000987000	CXCL10, CCL3, HGF
cytokine activity (GO:0005125)	233	3	0.000050200	0.049200000	CXCL10, CCL3, OPG
receptor ligand activity (GO:0048018)	493	4	0.000011100	0.027100000	CXCL10, CCL3, OPG, HGF
signaling receptor activator activity (GO:0030546)	500	4	0.000011700	0.019100000	CXCL10, CCL3, OPG, HGF
signaling receptor regulator activity (GO:0030545)	549	4	0.000016900	0.020600000	CXCL10, CCL3, OPG, HGF
signaling receptor binding (GO:0005102)	1591	5	0.000051000	0.041600000	CXCL10, CCL3, OPG, HGF, ITGA11
Results obtained from PANTHER functional classification platform (http://www.pantherdb.org)					

Supplementary Table 20. Protein-Protein interaction network analyses powered by STRING database

Node 1	CCL3	CCL3	CCL3	CXCL10	CXCL10	HGF	HGF	OPG
Node 2	HGF	OPG	CXCL10	HGF	CCL3	CCL3	CXCL10	CCL3
Node 1 String ID	9606.ENSP000000477908	9606.ENSP00000477908	9606.ENSP00000477908	9606.ENSP00000305651	9606.ENSP00000305651	9606.ENSP00000222390	9606.ENSP00000222390	9606.ENSP00000297350
Node 2 String ID	9606.ENSP000000222390	9606.ENSP00000297350	9606.ENSP00000305651	9606.ENSP00000222390	9606.ENSP00000477908	9606.ENSP00000477908	9606.ENSP00000305651	9606.ENSP00000477908
Neighborhood on Chromosome	0	0	0	0	0	0	0	0
Gene Fusion	0	0	0	0	0	0	0	0
Phylogenetic Oooccurrence	0	0	0	0	0	0	0	0
Sequence Homology	0	0	0	0	0	0	0	0
CoExpression	0.065	0	0.237	0.066	0.237	0.065	0.066	0
Experimentally Determined Interaction	0	0	0	0	0	0	0	0
Co-Association in Curated Databases (e.g., KEGG)	0	0	0.9	0	0.9	0	0	0
Co-Annotation (PubMed Abstracts)	0.473	0.451	0.906	0.501	0.906	0.473	0.501	0.451
Combined Score	0.486	0.451	0.992	0.514	0.992	0.486	0.514	0.451
number of nodes: 7								
number of edges: 4								
average node degree: 1.14								
avg. local clustering coefficient: 0.476								
expected number of edges: 0								
PPI enrichment p-value: 0.000776*								
* Proteins have more interactions among themselves than what would be expected for a random set of proteins of the same size and degree distribution drawn from the genome. Such an enrichment indicates that the proteins are at least partially biologically connected, as a group.								
Results obtained from protein interaction networks assessed using STRING (https://string-db.org)								

**Supplementary Table 21.** Therapeutic drugs targeting candidate proteins of interest									
Target Protein Information									
Full Protein Name	Protein Name	Drug ID	Drug Name						Disease Target
				Preclinical Phase			Investigative	Discontinued	
				3	2	1			
Macrophage inflammatory protein 1-alpha	CCL3	D02PMT	Nagrestipen			X			Solid Tumor/Cancer; Radiation Oncology
C-X-C motif chemokine 10	CXCL10	D0L7LH	Anti-IP10		X				Ulcerative Colitis
C-X-C motif chemokine 10	CXCL10	D00YWL	BMS-936557		X				Immune System Disease
C-X-C motif chemokine 10	CXCL10	DOVE3U	JT02		X				Inflammatory Bowel Disease
C-X-C motif chemokine 10	CXCL10	D0C1UF	MDX-1100		X				Crohn's Disease
C-X-C motif chemokine 10	CXCL10	D03VNB	NI-0801		X				Autoimmune Diabetes
C-X-C motif chemokine 10	CXCL10	D1A8NU	NG-641			X			Solid Tumor/Cancer
C-X-C motif chemokine 10	CXCL10	D0F6OK	N-Methylleucine				X		
Hepatocyte growth factor	HGF	D9CI7V	Donaperminogene seltoplasmid	X					Diabetic Foot Ulcer
Hepatocyte growth factor	HGF	D0D4OS	VM-202	X					Angina Pectoris
Hepatocyte growth factor	HGF	D03UBW	Ficlatuzumab		X				Non-small-cell lung cancer
Hepatocyte growth factor	HGF	D0ME7Q	MP0250		X	X			Non-small-cell lung cancer

Hepatocyte growth factor	HGF	D0P4KU	ABI-011			X			Solid tumour/cancer
Hepatocyte growth factor	HGF	D0H9HT	TAK-701			X			Advanced Malignancy
Hepatocyte growth factor	HGF	D07AOO	Rilotumumab		X			X	Grade IV malignant glioma
Hepatocyte growth factor	HGF	D0B0SV	N,O6-Disulfo-Glucosamine				X		
Hepatocyte growth factor	HGF	D06VYT	O2-Sulfo-Glucuronic Acid				X		
Nuclear Factor Of Activated T Cells 3	NFATC3	NONE	NONE						
Integrin alpha-11	ITGA11	NONE	NONE						
CUB domain-containing protein 1	CDCP1	NONE	NONE						
Osteoclastogenesis inhibitory factor	TNFRSF11B	NONE	NONE						
Results obtained from The Therapeutic Target Database (http://db.idrblab.net/ttd/)									
